# TAGLN2 polymerizes G-actin in a low ionic state but blocks Arp2/3-nucleated actin branching in physiological conditions

**DOI:** 10.1038/s41598-018-23816-2

**Published:** 2018-04-03

**Authors:** Hye-Ran Kim, Min-Sung Kwon, Sangmin Lee, YeVin Mun, Kyung-Sik Lee, Chang-Hyun Kim, Bo-Ra Na, Bit Na Rae Kim, Indre Piragyte, Hyun-Su Lee, Youngsoo Jun, Mi Sun Jin, Young-Min Hyun, Hyun Suk Jung, Ji Young Mun, Chang-Duk Jun

**Affiliations:** 1 School of Life Sciences, GIST, Gwangju, 61005 Korea; 2 Immune Synapse and Cell Therapy Research Center, GIST, Gwangju, 61005 Korea; 3 World Institute of Kimchi, Gwangju, 61755 Korea; 4 Department of Biochemistry, College of Natural Sciences, Kangwon National University, 1, Kangwondaehak-gil, Chuncheon-si, Gangwon-do 24341 Korea; 5 Department of Anatomy, Yonsei University College of Medicine, Seoul, 03722 Korea; 6 Department of Biomedical Laboratory Science, College of Health Science, Eulji University, Seongnam-si, Gyeonggi-do 13135 Korea; 7 Present Address: Department of Structure and Function of Neural Network, Korea Brain Research Institute, Dong-gu, Daegu Korea

## Abstract

TAGLN is an actin-binding protein family that comprises three isoforms with theorized roles in smooth muscle differentiation, tumour development, lymphocyte activation, and brain chemistry. However, their fundamental characteristics in regulation of the actin-based cytoskeleton are not fully understood. Here we show that TAGLN2 (including TAGLN1 and TAGLN3) extensively nucleates G-actin polymerization under low-salt conditions, where polymerization would be completely suppressed. The calponin homology domain and actin-binding loop are essential to mechanically connect two adjacent G-actins, thereby mediating multimeric interactions. However, TAGLN2 blocked the Arp2/3 complex binding to actin filaments under physiological salt conditions, thereby inhibiting branched actin nucleation. In HeLa and T cells, TAGLN2 enhanced filopodium-like membrane protrusion. Collectively, the dual functional nature of TAGLN2—G-actin polymerization and Arp2/3 complex inhibition—may account for the mechanisms of filopodia development at the edge of Arp2/3-rich lamellipodia in various cell types.

## Introduction

The TAGLN superfamily comprises TAGLN1, 2, and 3 isoforms, which have high degrees of sequence identity (~80%). TAGLN1 (also known as smooth muscle protein 22α or SM22α), which is an abundant, smooth muscle-specific 22-kD protein that serves as an early marker of smooth muscle tissue, is the best characterized^1^. TAGLN2 (also known as SM22β) is predominantly expressed in lymphocytes and certain non-smooth muscle cells^2^. Recently, our group revealed that TAGLN2 is also greatly induced by lipopolysaccharide (LPS)—a ligand for TLR4—in peritoneal and bone marrow-derived macrophages^3^. TAGLN3 (also known as neuronal protein 22, NP22, or NP25) is specifically expressed in brain tissue and upregulated in the superior frontal cortex and hippocampus in chronic alcoholic humans and rats^4,5^.

TAGLN was first discovered in chicken gizzard smooth muscle^6^ and was later named ‘transgelin’ because of its transformation-sensitive and rapid actin-gelling properties^7^. Indeed, the principal roles of TAGLN proteins in several cellular processes—including cell migration, apoptosis, differentiation, and tumour progression—are associated with its actin-binding and cytoskeleton-stabilizing properties^8^. For example, TAGLN1 maintains the differentiated phenotype of vascular smooth muscle cells (VSMCs) by inducing filamentous actin bundling^9^. TAGLN2 in T cells stabilizes cortical F-actin to maintain the immunological synapse which then allows effector T cells to efficiently kill virus-infected cells^2^. TAGLN2 is also involved in membrane ruffling and augments phagocytic function in macrophages^3^. TAGLN3 colocalizes with both cytoskeletal microtubules and microfilaments in neurite-like processes^8^, and transfection with mutant TAGLN3 containing a deletion of the putative actin-binding domain fails to induce process formation. The yeast transgelin homolog (Scp1) induces actin bundling and regulates stability and organization of the actin cytoskeleton^10^. However, the fundamental characteristics of TAGLN in regulation of the actin-based cytoskeleton have still not been fully addressed.

In the present study, we investigated the unknown roles of TAGLNs in regulation of the actin cytoskeleton. We surprisingly observed that TAGLN2 directly polymerizes globular (G)-actin in low-salt conditions in which actin polymerization would be completely suppressed. G-actin polymerizes spontaneously in high-salt conditions *in vitro*. It is therefore believed that in living cells where the ionic conditions are optimal, actin polymerization is spatially and temporally regulated by several actin-regulating proteins. However, only a few proteins can induce actin polymerization under low-salt conditions^11–17^. This newly found characteristic of TAGLN2 led us to determine the actin-based properties of full-length TAGLN2 and its subdomains. We found that TAGLN family members share similar characteristics. On the other hand, TAGLN2 blocked the binding of the Arp2/3 complex to the actin filament under physiological salt conditions. These characteristics of TAGLN2 may be associated with its biological functions to induce filopodia-like phenotypes. We therefore determined the localization and function of TAGLN2 in HeLa cells and human and mouse T cells, as these cells express considerable amounts of TAGLN2.

## Results

### TAGLN2 binds to and polymerizes G-actin in low-salt conditions

**Figure 1:**
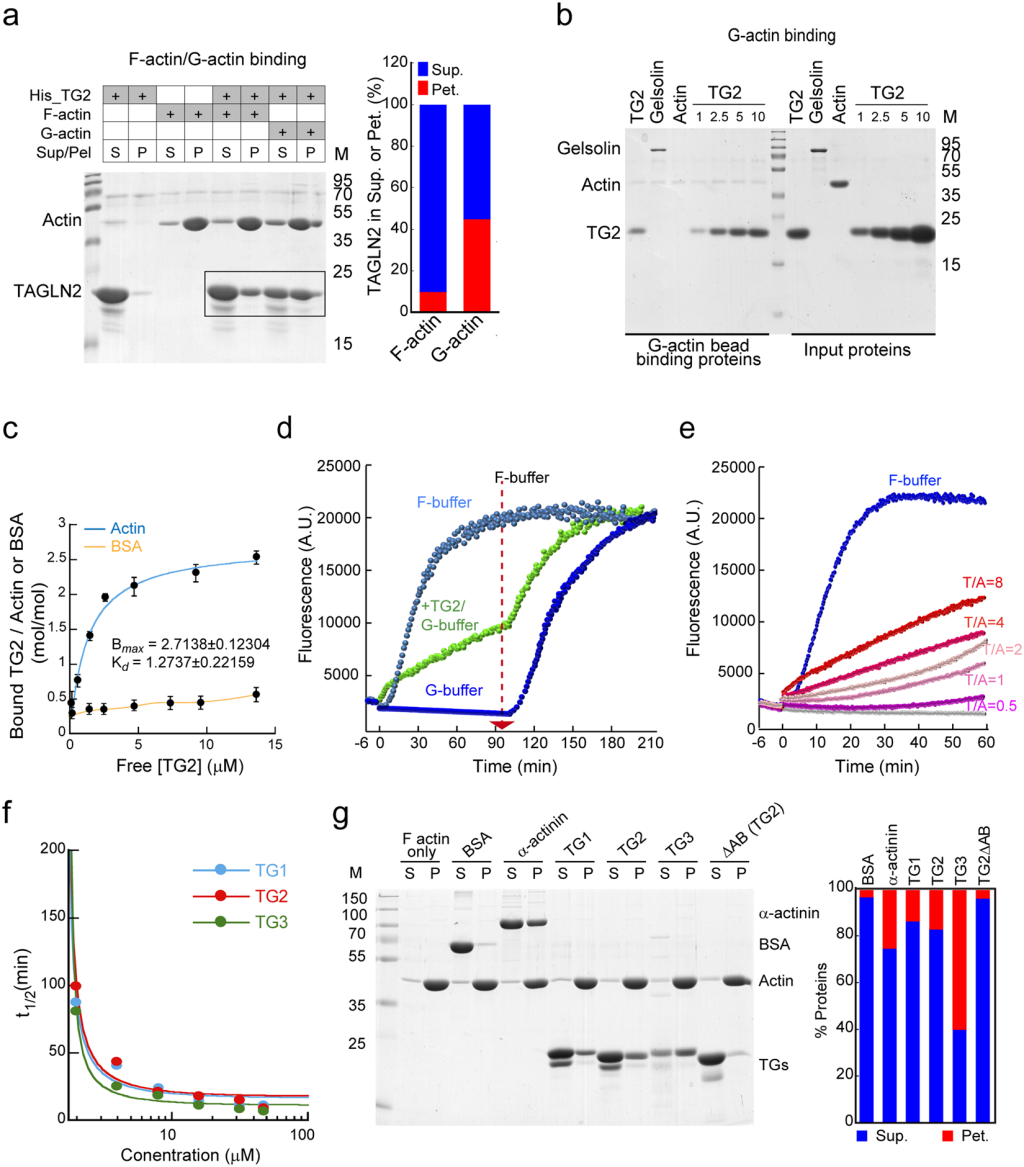
TAGLN2 polymerizes G-actin in low-salt buffer. (**a**) TAGLN2 (TG2) binding to pre-polymerized F-actin or G-actin was assessed after incubation for 30 min. S, supernatant; P, pellet. Full-length blots/gels are presented in Supplementary Fig. S5. (b) Actin beads were incubated with gelsolin (2.5 μg), actin (2.5 μg), and increasing amounts of TAGLN2 (1, 2.5, 5, 10 μg) in G-buffer for 60 min. The samples were assessed for G-actin binding activity as described in the Methods. Full-length blots/gels are presented in Supplementary Fig. S5. (**c**) Affinity of TAGLN2 to G-actin was measured as described in the Methods. Blue line, binding to G-actin; orange line, binding to BSA. (**d**–**f**) Time-based fluorometric analysis of pyrene-labelled actin polymerization. Reactions were as follows: (**d**) presence or absence of TAGLN2 in low-salt G-buffer or high-salt F-buffer (arrow, time of F-buffer addition); (**e**) increasing concentrations of His-TG2 in G-buffer (T/A = TG2/actin); (**f**) increasing concentrations of TAGLN1, 2, and 3. Time to half-maximum fluorescence (t_1/2_) at each TAGLN concentration was plotted. Results are representative of at least three independent experiments; (**g**) analysis of pre-polymerized F-actin binding affinity of TAGLN2 family members and TG2ΔAB. The percentage of total actin in the pellet was quantified (right). S, supernatant; P, pellet. Full-length blots/gels are presented in Supplementary Fig. S5.

TAGLN family members are F-actin-binding proteins that induce actin bundling^6^ and localize at F-actin-rich regions such as stress fibres in cells^8^. However, the biochemical properties of TAGLN in regulation of the actin cytoskeleton are still not fully understood. We therefore initially investigated whether TAGLN2 has any other uncharacterized property in terms of actin control. Using high-speed co-sedimentation assay, we unexpectedly observed that TAGLN2 also binds to G-actin, and interestingly, based on the recovery of TAGLN2 + G-actin in the F-actin fraction (Fig. 1a), addition of TAGLN2 to G-actin produced filamentous forms of actin under low-salt conditions in which actin polymerization is suppressed, suggesting that TAGLN2 may nucleate G-actin polymerization under low-salt conditions. To further confirm the actual binding to G-actin, TAGLN2 was incubated with G-actin-coated sepharose beads. As shown in Fig. 1b, TAGLN2 bound to G-actin in a dose-dependent manner. To further determine the binding affinity of TAGLN2 to G-actin, we performed co-sedimentation assay with free G-actin. BSA was used as a negative control. The binding of TAGLN2 to actin monomers was saturated at a 2.5:1 ratio in low-salt G-buffer (B_*max*_ = 2.8817 ± 0.072028 mol/mol) with *K*_d_ of 0.921 μM (Fig. 1c). Although buffer conditions were different, this result suggested that the binding affinity of TAGLN2 to G-actin in G-buffer (CaCl_2_ = 0.2 mM) is much higher than that of F-actin which we determined previously (B_*max*_ = 0.915 ± 0.102 mol/mol, *K*_d_ of 7.39 μM) in high-salt F-buffer (KCl = 50 mM and MgCl_2_ = 2 mM)^2^. We further confirmed the TAGLN2-mediated G-actin polymerization by pyrene-based actin polymerization assay. TAGLN2-dependent polymerization was unambiguously observed in a dose-dependent manner (Fig. 1d and e). No initial lag phase, which was seen in spontaneous polymerization, was observed. To rule out the potential effect of the 6 × His tag, which is known to contain a high positive charge, on the actin polymerization, we purified TAGLN2 protein from the His-TAGLN2 complex by thrombin treatment (Supplementary Fig. S1a, *left*). The purified TAGLN2 was further confirmed by western blot (Supplementary Fig. S1a, *right*). We performed actin polymerization assay in G- or F-buffer conditions with purified TAGLN2 proteins and verified that actin polymerization in G-buffer was due to TAGLN2 itself and not the His tag (Supplementary Fig. S1b).

The TAGLN1 and TAGLN3 isoforms also induced G-actin polymerization in a similar manner (Fig. 1f), suggesting that all three isoforms were equally active in this regard. Specifically, TAGLN2 activity was approximate to that of TAGLN1 under identical conditions but slightly lower than that of TAGLN3, consistent with data showing a lower binding affinity of TAGLN2 to actin than that of TAGLN3 (Fig. 1f and g). The secondary structures revealed that TAGLN3 has more positively charged amino acids around the actin-binding site (153–163) than TAGLN1 and 2. Consistently, deletion of the actin-binding site (ΔAB) resulted in significant loss of actin binding affinity (Fig. 1g), suggesting that electrostatic interaction, at least partially, is important for G-actin polymerization.

**Figure 2:**
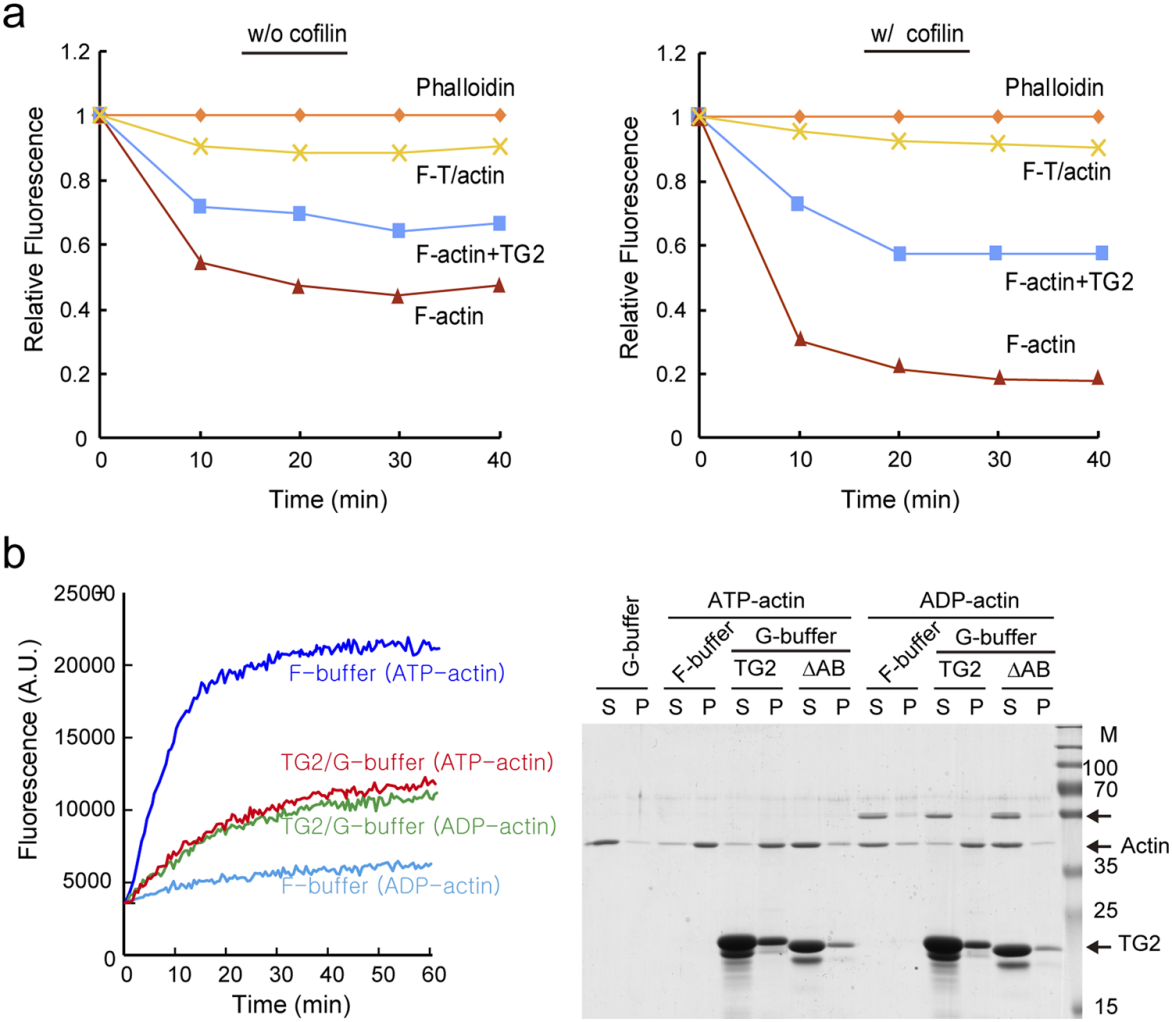
TAGLN2-polymerized F-T/actin complex is resistant to depolymerization and has no preference between ATP-actin and ADP-actin. (**a**) Actin polymerization reactions containing actin and TAGLN2 (actin + TAGLN2) or actin alone (actin) in G-buffer and actin and TAGLN2 (actin + TAGLN2) or actin and phalloidin (actin + phalloidin) in F-buffer were diluted fivefold with G-buffer to initiate depolymerization in the absence (left) or presence (right) of cofilin. Results are representative of at least three independent experiments. (**b**) TAGLN2 uses both ATP-actin and ADP-actin for polymerization in G-buffer as monitored by fluorescence (left) or co-sedimentation (right). Results are representative of at least three independent experiments. Full-length blots/gels are presented in Supplementary Fig. S6.

Previously, we reported that TAGLN2 stabilizes F-actin and also blocks cofilin-mediated actin depolymerization^2^. We tested whether the stability of the TAGLN2/actin (F-T/actin) complex is stronger than that of F-actin or F-actin decorated with TAGLN2 (F-actin + TG2) in the presence or absence of cofilin. Notably, T/actin was stable and highly resistant to the cofilin-mediated depolymerization (Fig. 2a). Because actin has ATPase activity^18^, we next determined the ATP requirement for TAGLN2-driven G-actin polymerization. ADP-actin can also polymerize^19^ but at a much lower extent than ATP-actin^20^. Interestingly, exchanging ATP-actin with ADP-actin in F-buffer significantly reduced the polymerization rate, yet TAGLN2-mediated ADP-actin polymerization was not affected, as determined by pyrene-based actin polymerization and high-speed co-sedimentation assays (Fig. 2b), demonstrating that TAGLN2-driven G-actin polymerization is independent of ATP and molecular affinity between TAGLN2 and actin is likely more important.

### Visualization of TAGLN2-mediated G-actin polymerization in low-salt conditions

**Figure 3:**
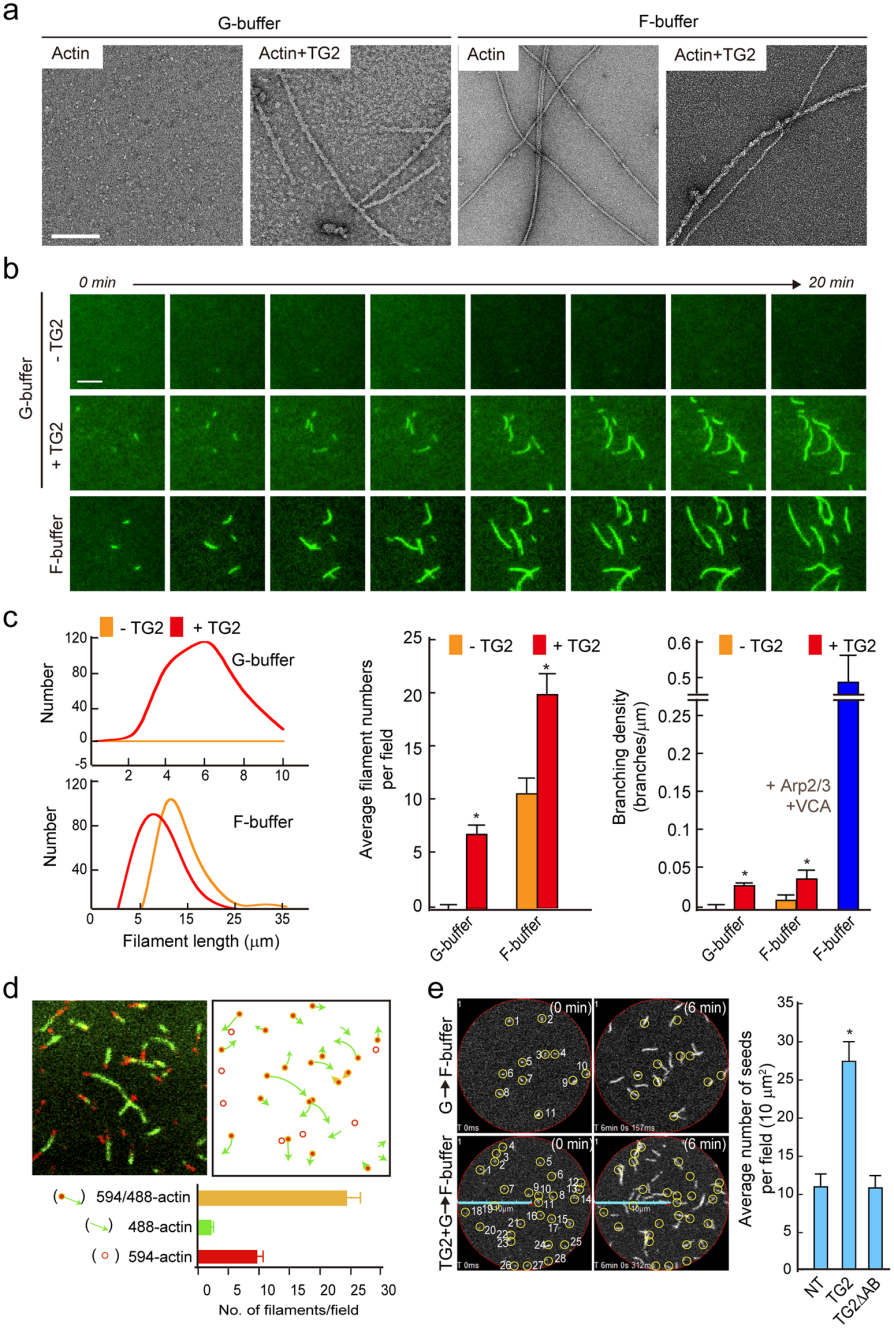
Visualization of TAGLN2-mediated G-actin polymerization in low-salt buffer. (**a**) Electron micrographs of negatively stained actin polymers in G- and F-buffer. Left column represents the control images showing G-actin and the distinctive appearances of F-T/actin complex in G-buffer. Right column shows filamentous (F) actin and TAGLN2 and F-actin complex in F-buffer. Scale bars, 2 μm. (**b**) Real-time actin assembly in the absence or presence of TAGLN2 in G-buffer was monitored by TIRF microscopy using Atto488-actin. Scale bars, 2 μm. (**c**) Quantification of average filament length, average filament numbers per field, and branching density in each condition. *p < 0.05 *versus* without TG2. (**d**) TAGLN2 (0.4 μM)-Atto594 actin (0.2 μM) complex was formed in the G-buffer condition, diluted 1/10 in G-buffer, and loaded onto an NEM-coated coverglass. Mg^2+^-exchanged Ca^2+^-Atto488 actin (0.2 μM) mixture in F-buffer was then loaded onto the coverglass for 10 min and viewed by confocal microscopy. Results are representative of at least three independent experiments. (**e**) Time-lapse imaging of actin growth from TAGLN2-Atto488 actin seed. The number of actin seeds was increased in the presence of full-length TAGLN2 but not TAGLN2ΔAB. *p < 0.05 *versus* NT.

To visualize the F-T/actin complexes, we next conducted experiments using transmission electron microscopy (TEM) and total internal reflection fluorescence (TIRF) microscopy. TAGLN2 polymerized G-actin to form filamentous TAGLN/actin (F-T/actin) fibres (Fig. 3a) which were wider than those of F-actin alone (~15 nm vs. ~9 nm, respectively). Similar structures were also seen in F-buffer containing TAGLN2, suggesting that TAGLN2 mainly contributes to the wider appearance of F-T/actin complexes (Fig. 3a). Time-lapse studies with TIRF microscopy (Fig. 3b) and confocal microscopy (See Supplementary Videos S1–S4) also showed that F-T/actin elongates faster at one end, indicative of barbed-end polymerization. Moreover, the lengths of F-T/actin fibres were shorter than those of fibres made with actin alone or actin plus TAGLN2 in F-buffer (Fig. 3c). The numbers of F-T/actin filaments were lower in G-buffer than those of actin alone or actin plus TAGLN2 in F-buffer, presumably because of a slow binding rate of TAGLN2/actin to the growing ends of F-T/actin (Fig. 3c). Some F-T/actins showed actin branches (Fig. 3c); however, the range of angles was not indicative of a specific interaction like that of Arp2/3 complex, presumably because of TAGLN2 binding on the side of F-T/actin stalk (Fig. 3a). Interestingly, these analyses also demonstrated that filament number was inversely proportional to length in F-buffer (Fig. 3c, *middle*), in which filaments made in the presence of TAGLN2 were shorter than those made with actin alone (Fig. 3c, *left*), suggesting that TAGLN2 may generate more F-T/actin seeds in physiological salt conditions. To prove this, we performed the following experiments. First, TAGLN2 was incubated with Alexa594-labelled G-actin in G-buffer for 5 min, and then the complex was further incubated with Alexa488 G-actin in F-buffer. We unambiguously observed that the green filament was grown from red actin, suggesting that F-T/actin structure can provide actin seeds (Fig. 3d). Second, after exchange of buffers from G to F, the number of actin filaments was monitored by time-lapse imaging analysis, and we found that TAGLN2 significantly increased the number of actin filaments (Fig. 3e). Taken together, these results demonstrated that TAGLN2 has actin-nucleating activity, albeit the activity is less than that of actin nucleation factors such as formin and Spir^21,22^.

### Three-dimensional reconstruction of F-T/actin reveals that TAGLN2 serves as a molecular staple

**Figure 4:**
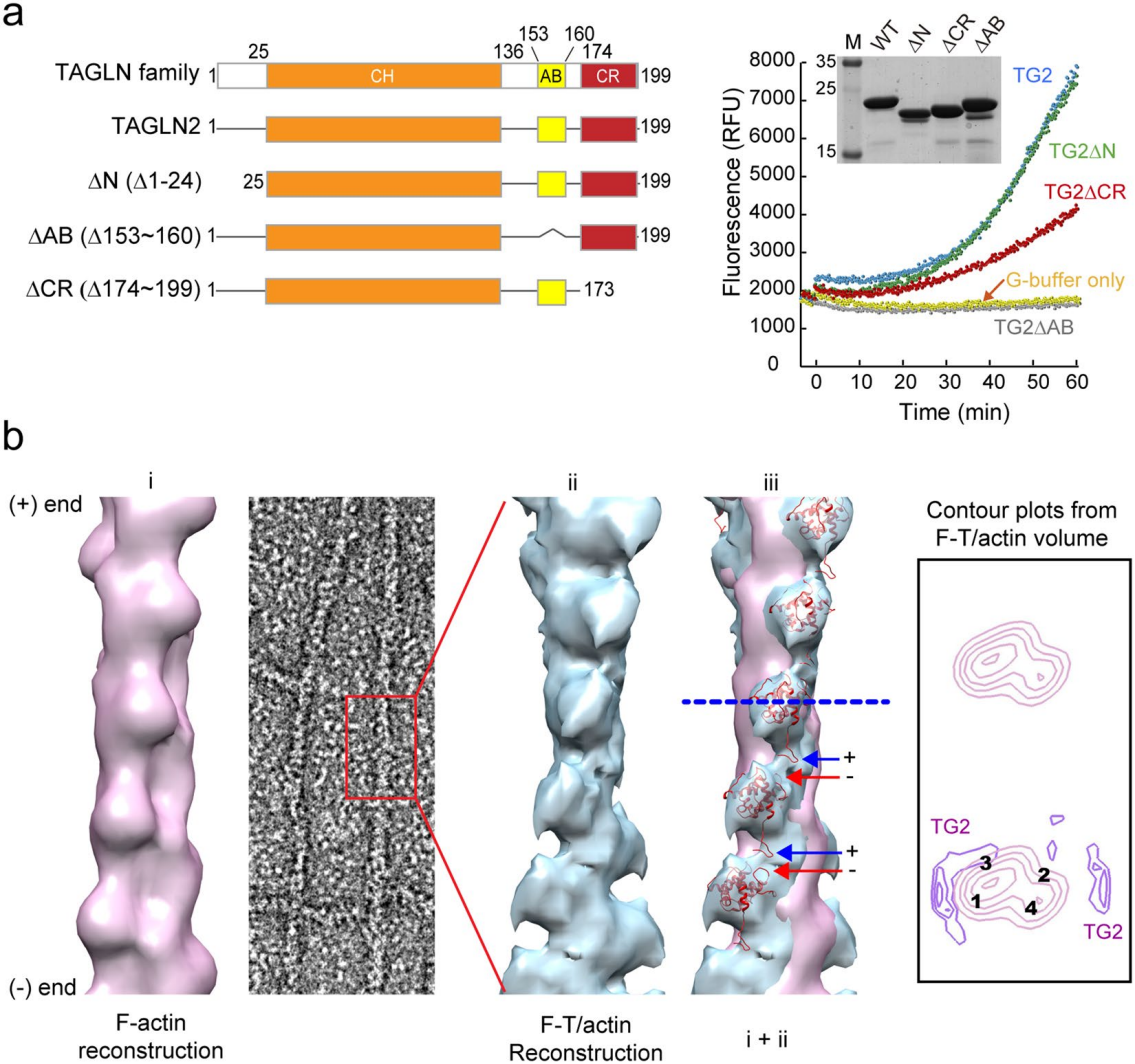
Three-dimensional reconstruction of F-T/actin reveals that TAGLN2 serves as a molecular staple. (**a**) Identification of essential actin-binding regions of TAGLN2. Schematic diagram of the TAGLN2 constructs (left) and fluorometric analysis of pyrene-labelled actin polymerization in the presence of the indicated proteins [2 μM actin, 8 μM TAGLN2 (TG2) or TG2 mutants, 20 nM Arp2/3, and 200 nM GST_VCA] (right) are shown. Purified proteins were stained with Coomassie blue (right). Full-length blots/gels are presented in Supplementary Fig. S6. (**b**) Three-dimensional (3D) reconstruction of TAGLN2/actin filaments (F-T/actin). i, Surface view of 3D F-actin reconstruction was generated with IHRSR using actin as a reference. Actin subdomains are labelled as 1–4. ii, 3D reconstruction of F-T/actin complex. iii, Superimposition of F-T/actin reconstruction (cyan) and the TAGLN2 atomic model (PDB ID: 1WYM, red) was fitted into extra densities existing in the two superimposed models. Arrows point to additional density of decorated filaments. Filaments are oriented with pointed end at top. Numbers indicate actin SDs. Transverse sections of actin and F-T/actin reconstruction showing actin subdomains and positioning of TAGLN2, respectively (arrows).

TAGLN family members contain a single CH domain, AB motif, and a C-terminal calponin-like repeat (CR) region (Fig. 4a and see Supplementary Fig. S2). To identify essential regions that mediate G-actin polymerization, we constructed TAGLN2 deletion mutants and tested their activity in terms of G-actin polymerization. These analyses revealed that the first 25 N-terminal residues before the CH domain and the last C-terminal CR regions are not essential for G-actin polymerization (Fig. 4a). Structurally, TAGLNs belong to the calponin protein family^23^. Although a previous report demonstrated that the CH domain alone does not mediate actin binding ^23^, our study suggested that it is necessary to recruit opposite actin units and stabilize the TAGLN2-actin structure in concert with the AB motif (Fig. 4a).

We expected that G-actin polymerization by TAGLN2 is probably due to its ability to stabilize the formation of short segments that would otherwise be highly unstable. To characterise the structural nature of the F-T/actin complex, well-preserved regions of negatively stained filaments showing a wide variety of appearances were selected (Fig. 4b). Their three-dimensional (3D) structures were determined by single-particle image analysis based on the iterative helical real space reconstruction (IHRSR) approach^24^ to reveal how TAGLN2 interacts with G-actin (Fig. 4b). Some actin filaments were clearly bundled structures decorated with TAGLN2 (data not shown). However, numerous well-separated filaments showed slight increases in diameter compared with that of control F-actin (~9 nm) (Fig. 4b). F-T/actin complex showed lower contrast and higher background than those of the undecorated F-actin control, presumably due to the presence of excess unbound fragment. Nevertheless, the F-T/actin complex was clear based on repeated protrusions. 3D reconstructions were further carried out on selected straightened filaments of F-actin alone and F-T/actin complex. Reconstruction of F-actin showed the well-known arrangement of four subdomains (SDs) in each actin subunit, with SD1 and SD2 on the outside of the filament and SD3 and SD4 near its centre (Fig. 4b). Filaments combined with TAGLN2 revealed a higher density of TAGLN2 on actin SD1 and SD3 (Fig. 4b). To better understand the binding site, we fitted an atomic model of F-actin and TAGLN2 (Protein Data Bank [PDB] code: 1WYM) to the 3D reconstructions of F-actin and F-T/actin (Fig. 4b). The putative fitting of atomic structure effectively represented the hypothesis we originally expected in which TAGLN2 could affect the stability of tentatively polymerized actin structure that otherwise would be unstable. The negatively charged aspartate or glutamate (red arrow) in the C-terminal’s protrusion of TAGLN2 and the positively charged lysine (blue arrow) in the N-terminal showed the possibility of ionic interaction between two TAGLN2 molecules in close distance. Transverse sections (blue dotted line) of the reconstructions (F-actin only and F-actin subtracted from F-T/actin) clearly showed the extra density on SD1 and SD3 in F-T/actin complex. From this comparison, we revealed that TAGLN2 serves as a molecular staple by binding SD1 and 3 on neighbouring actin molecules.

### TAGLN2 blocks Arp2/3 complex-nucleated actin branching in high-salt conditions

**Figure 5:**
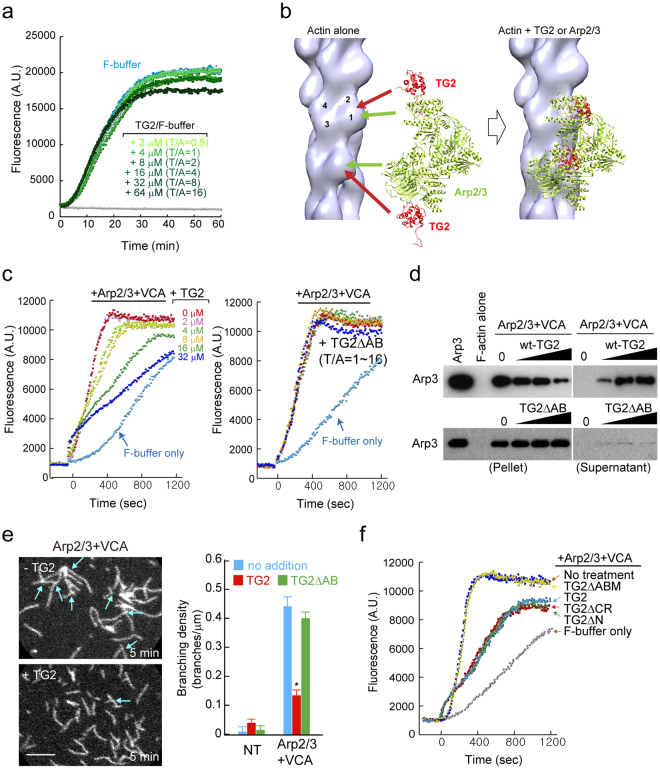
TAGLN2 inhibits Arp2/3 complex-mediated nucleation of branched filaments. (**a**) Time-based fluorometric analysis of pyrene-labelled actin polymerization. Polymerization assay in presence of His-TG2 (0, 2, 4, 8, 16, 32, and 64 μM) in F-buffer. Results are representative of at least three independent experiments. (**b**) Fitting of TG2 (red) and Arp2/3 (green) to F-actin reconstruction, showing the clash between TG2 and ARP2/3. (**c**) Dose-dependent inhibition of Arp2/3 and VCA-stimulated actin nucleation by TAGLN2. (0–32 μM TAGLN2 [TG2] or TAGLN2ΔAB, 20 nM Arp2/3, 200 nM GST_VCA). Results are representative of at least three independent experiments. (**d**) Competition with the Arp2/3 complex for actin binding in the presence of TAGLN2 or TG2ΔAB. Full-length blots/gels are presented in Supplementary Fig. S7. (**e**) Real-time imaging of actin branching formed by Arp2/3 and VCA with/without TAGLN2 (left). Scale bars, 2 μm. Quantification of branching density for each condition (right). *p < 0.05 *versus* no addition. (**f**) Fluorometric analysis of pyrene-labelled actin polymerization in the presence of the indicated proteins (Note, 2 μM actin, 8 μM TAGLN2 [TG2] or TAGLN2 mutants; 20 nM Arp2/3, and 200 nM GST_VCA). Results are representative of at least three independent experiments.

We next determined the biochemical properties of TAGLN2 under physiological buffer conditions. We first questioned whether TAGLN2 increases/decreases the kinetics of spontaneous actin polymerization under physiological buffer conditions. However, TAGLN2 only slightly reduced spontaneous actin polymerization at higher concentration (64 μM; Fig. 5a). Thus, it is unlikely that TAGLN2 affects the kinetics of actin polymerization in cells. A previous report demonstrated that actin side-binding proteins such as EPLIN prevent secondary activation of nucleation mediated by Arp2/3 complex^25^. As TAGLN2 also acts as a side-binding protein, we next addressed whether TAGLN2 influences the binding of Arp2/3 complex to actin filaments. Notably, the location of the TAGLN2 binding site in actin overlapped with the Arp 2/3 actin binding site^26^ and would interfere with the binding of Arp2/3 complex to actin (Fig. 5b). WT TAGLN2, but not its actin-binding motif deletion mutant (TG2ΔAB), significantly blocked the Arp2/3 and VCA-mediated branched actin nucleation in a concentration-dependent manner (Fig. 5c), as a consequence of the competition with Arp2/3 binding to F-actin in F-buffer (Fig. 5d). We observed the generation of fewer actin branched junctions in the presence of TAGLN2 (Fig. 5e and Supplementary Videos S5 and S6). We also found that both the CH domain and actin-binding motif are critical to block Arp2/3-mediated actin nucleation (Fig. 5f).

### TAGLN2 is involved in filopodia protrusion in various cell types

Filopodia are long, actin-rich cellular protrusions on the leading edge of many motile cells, including fibroblasts, nerve growth corns, and immune cells^27–29^. The mechanisms of actin filament elongation and turnover in leading-edge filopodia are fairly well defined; however, the main issues, that is, how filopodia are initiated and how Arp2/3 is excluded from the filopodium stalk, remain a matter of debate. Interestingly, the binding pattern utilised by TAGLN2 to polymerize G-actin under low-salt conditions was highly similar to that of *Salmonella* invasion protein A (SipA)^16,30^, while the structural similarity was not found (see Supplementary Fig. S3). Previous reports demonstrated that the SipA^446–684^ fragment polymerizes G-actin in low-salt conditions, resulting in pronounced outward extension of *Salmonella*-induced membrane ruffles and filopodia that facilitate bacterial uptake^16,30^. Thus, we considered whether TAGLN2 might be involved in filopodium initiation and/or elongation. This idea was further supported by the fact that TAGLN2 interferes with the binding of Arp2/3 complex to actin.

**Figure 6:**
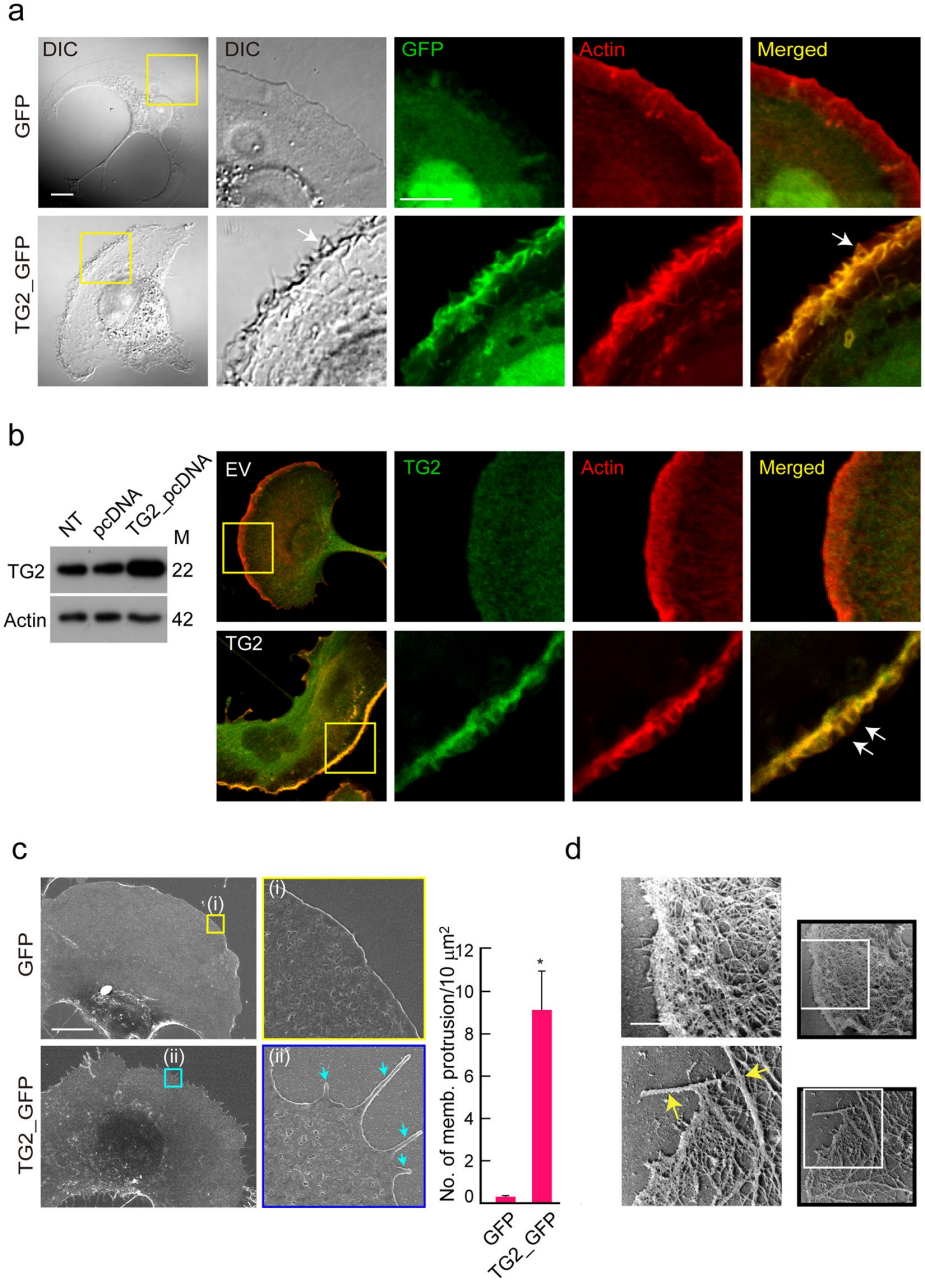
TAGLN2 mediates filopodium formation in COS-7 cells. (**a**) Membrane protrusions in COS-7 cells expressing GFP or TG2_GFP stimulated with PMA. Scale bar, 5 μm. White arrows indicate spiky-like membrane protrusions at lamellipodia. (**b**) Expression and localization of WT TG2 in COS-7 cells. COS-7 cells were transfected with non-GFP-tagged WT TG2 and analysed as described in (**a**). Full-length blots/gels are presented in Supplementary Fig. S7. (**c** and **d**) COS-7 cells expressing GFP or TG2_GFP were stimulated with PMA and imaged by scanning EM (**c**) and platinum replica EM (**d**). Blue and yellow arrows indicate the edges of the cell membrane. Scale bar, 10 μm. *p < 0.05 *versus* GFP-transfected cells.

**Figure 7:**
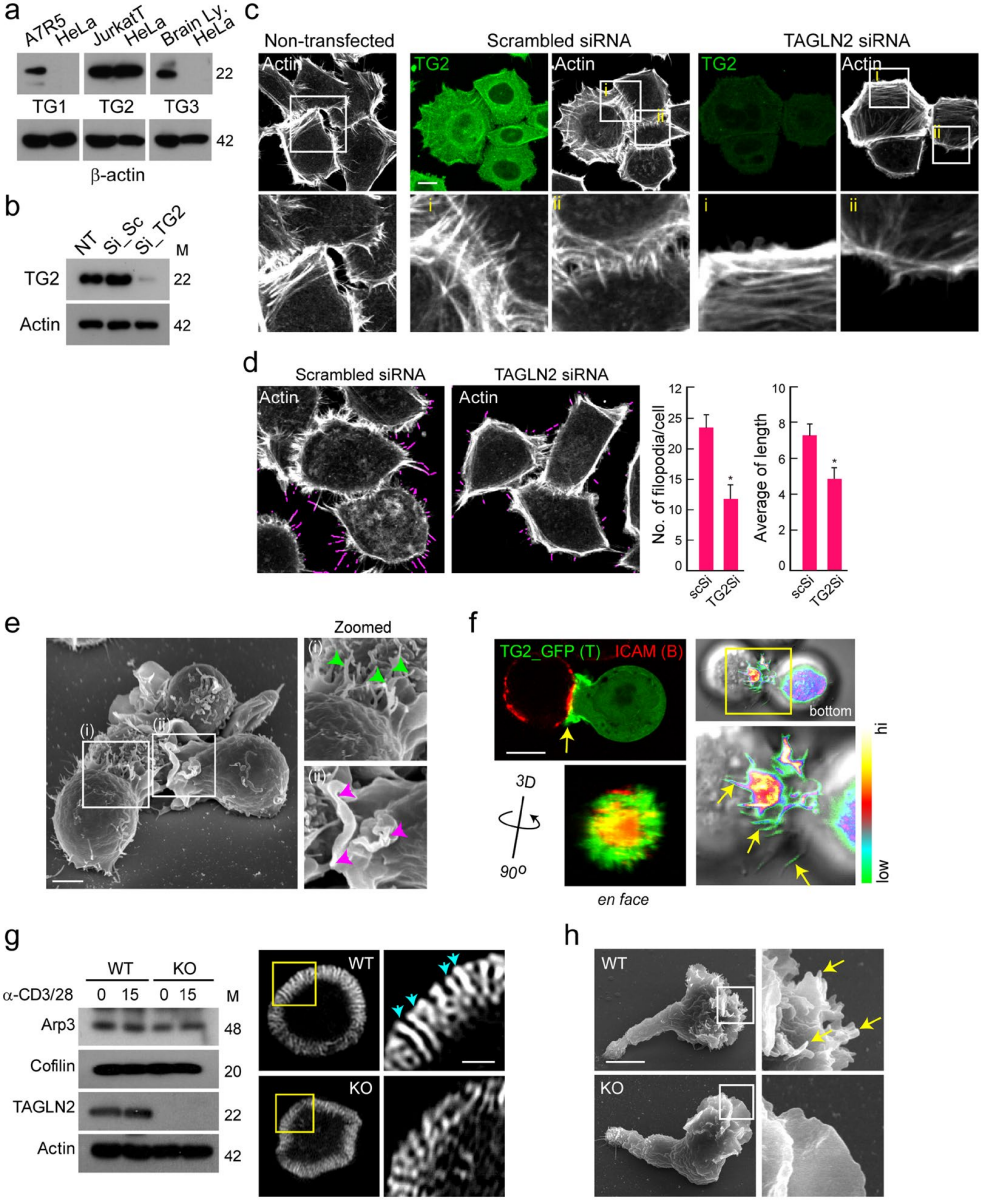
Knockdown of TAGLN2 reduces filopodia in HeLa cells and mouse T cells. (**a**) TAGLN1, 2, and 3 expression in HeLa cells with A7R5 cells, Jurkat T cells, and brain lysates as positive controls, respectively. Full-length blots/gels are presented in Supplementary Fig. S9. (**b**) TAGLN2 expression after siRNA knockdown in HeLa cells. Full-length blots/gels are presented in Supplementary Fig. S8. (**c**) Effect of TAGLN2 knockdown on filopodium formation in HeLa cells. (**d**) Quantification of filopodia and their average length by FiloQuant analysis. *p < 0.05 *versus* scrambled siRNA-transfected cells. (**e**) SEM micrographs recorded after 10-min co-incubation of Jurkat T cells and SEE-loaded Raji B cells. I, Elongated actin-rich filopodia projections (green arrow) were observed during antigen recognition in the early stage of the immunological synapse. ii, The purple arrow indicates the ruffled leading edge of a T cell forming a mature immunological synapse. (**f**) TG2_GFP-expressing Jurkat T cells were incubated for 20 min with SEE-loaded Raji B cells (red: ICAM1-Cy5), and then the cells were imaged by confocal microscopy. 3D rotating views (90 °C) of the top images are shown. Accumulation of TAGLN2 was revealed with pseudo-colour coded by fluorescent intensity. A rainbow scale was applied, with white representing the strongest expression. Scale bar, 10 μm. (**g**) Mouse CD3^+^ T cells obtained from WT and *TAGLN2*^*−/−*^ mice were subjected to western blotting against Arp3, cofilin, TAGLN2, and actin (left). Full-length blots/gels are presented in Supplementary Fig. S9. The cells were also placed on the anti-CD3-coated coverglass for 5 min, and the actin ring was further analysed with structured illumination microscopy (SIM) (right). The blue arrow indicates ordered actin structure. Boxed regions are shown enlarged at the right of the panel. Scale bar, 1 μm. (**h**) SEM images of representative CD3^+^ T blast from WT and *TAGLN2*^*−/−*^ mice on PLL-coated Si wafer. Boxed regions are shown enlarged at the right of the panel. The yellow arrow indicates finger-like microvilli generated in T cells. Scale bar, 2 μm.

We first determined the function of TAGLN2 in COS-7 cells. COS-7 cells transfected with GFP alone produced clear lamellipodia upon PMA stimulation^31^; however, those transfected with TAGLN2_GFP (TG2_GFP) generated spike-like filopodia at the lamellipodia (Fig. 6a). TG2_GFP signals localized to the lamellipodial region and strongly overlapped with F-actin (Fig. 6a). To rule out a potential effect of GFP fusion on cellular localization and generation of spike-like filopodia by TAGLN2, COS-7 cells were transfected with non-GFP-fused WT TAGLN2 and stained with anti-TG2 antibody. WT TAGLN2 was highly localized at the membrane protrusive regions, i.e. filopodia. Furthermore, WT TAGLN2, like TAGLN_GFP, also generated filopodia-like structures at the lamellipodia (Fig. 6b). Scanning EM analysis corroborated the presence of spike-like filopodia in these cells (Fig. 6c). EM of rotary-shadowed cells was also used to compare cytoskeletal organization in EV- and TG2-transfected cells (Fig. 6d). Spike-like actin bundles were clearly observed in TAGLN2-transfected COS-7 cells but not in EV-transfected cells (Fig. 6d). We next determined the effect of TAGLN2 depletion in cells that express considerable amounts of TAGLN2. We first utilised HeLa cells, as these cells have elevated TAGLN2 expression (Fig. 7a) and naturally develop highly ordered spike-like structures^32,33^. Interestingly, the siRNA-mediated knockdown of *TAGLN2*, but not treatment with scrambled siRNA, dramatically inhibited the spike-like protrusive structures in HeLa cells (Fig. 7b–d).

Previously, we found that among the three TAGLN isoforms, T cells only expressed TAGLN2, which excludes any potential redundant effects and enabled us to test the effect of TAGLN2 deficiency in T cells^2^. T-cell activation requires the plasticity of the actin cytoskeleton, which is based on the spatiotemporal regulation of the polymerization and depolymerization processes^29,34–36^. After initial contact with antigen presenting cells (APCs), T cells polarize many elongated filopodia (or microvilli) toward the APCs, which is presumably necessary to scan the surface for recognizable antigens and potential ligands on the APCs (Fig. 7e). We thus examined the cellular location of TAGLN2 in T cells. Interestingly, TAGLN2 signals were strongly concentrated at the filopodia-like membrane protrusive regions of the immunological synapse (Fig. 7f, arrowheads). We finally observed that *TAGLN2*^*−/−*^ T cells do not spread well on anti-CD3/28-coated coverslips compared with their WT counterparts (Fig. 7g). High-resolution microscopy showed that although TAGLN2 deficiency has no defects in Arp2/3 and cofilin proteins (Fig. 7g), these cells display impairments in ordered actin structure at the distal-supramolecular activation cluster when compared with their WT counterparts (Fig. 7g). We assumed that these ordered structures were actin bundles, which were recently revealed to propel T-cell receptor microcluster movement at the immune synapse^37^. Distortion of this structure may result in the instability of the immunological synapse^2^. We also examined the surfaces of activated T cells before T-cell interaction with antigen-pulsed B cells. WT T cells showed many small protrusions on the polarized surface, whereas *TAGLN2*^*−/−*^ T cells exhibited a mostly flat surface (Fig. 7h), suggesting that TAGLN2 accounts for filopodia formation during immunological synapse formation.

## Discussion

Actin is one of the most abundant proteins in all eukaryotic cells and participates in a variety of cellular processes including cell proliferation and differentiation, apoptosis, migration, and cellular signalling^36^. To accomplish the above processes, however, actin cytoskeletons must be spatiotemporally regulated by the actin-binding proteins (ABPs) that account for approximately 25% of total cellular protein^36^. TAGLN2 belongs to the ABP family and is known to regulate the actin cytoskeleton through actin binding^8^. However, biochemical characteristics of TAGLN2 and its family members have not been fully resolved. In the present study, we discovered novel biochemical characteristics of TAGLN2 and one interesting biological role that has not been reported to date. First, TAGLN family members directly bind to both G- and F-actin and extensively nucleate G-actin polymerization under low-salt conditions. In addition, TAGLN2 increases the number of actin seeds under physiological salt conditions. Second, both the CH domain and AB motif are essential for the TAGLN2-driven G-actin polymerization. Third, the TAGLN2/actin complex (F-T/actin) is resistant against cofilin-mediated actin depolymerization. Fourth, ATP-actin is not necessary for G-actin polymerization by TAGLN2 in low-salt conditions. Fifth, EM-based single-particle image analysis implied that TAGLN2 functions as a ‘molecular staple’ that binds to actin at one end and recruits another to mediate multimeric interactions. Sixth, TAGLN2 blocks Arp2/3 complex-nucleated actin branching in physiological salt conditions. Seventh, TAGLN2 induces filopodia-like membrane protrusion at the edge of lamellipodia in various cell types.

It is known that soluble fragments of myosin, caldesmon, and fesselin can induce polymerization of G-actin^14,15^. Moreover, poly-l-lysine and polyamines, such as spermine and spermidine, also cause transformation of G-actin to F-actin^15^. Hence, it is possible that the process of actin polymerization under low-salt conditions involves non-specific electrostatic interactions of poly-cations with negatively charged actin, which consequently abolishes repulsions between actin monomers. TAGLN2 contains highly positively charged actin-binding sites that may interact with actin in a manner similar to that of the poly-cations (Fig. S1). Therefore, it is plausible that F-actin formation in the absence of salt occurs, at least in part, through a condensation of oligomers stabilized by TAGLN2.

Fragments of the skeletal muscle proteins nebulin and myosin S-1 as well as the vinculin tail region are also known to bind actin and induce polymerization in low-salt conditions^11,38,39^. However, there are no indications that these fragments directly participate in actin polymerization in cells. The physiological roles of fesselin and caldesmon are also not clear. In fact, because G-actin polymerization does not naturally occur under low-salt conditions, it is hard to predict the *in vivo* physiology. Interestingly, LL-37, an antimicrobial peptide secreted from macrophages, polymorphonuclear leukocytes, and keratinocytes, induces G-actin polymerization—presumably via non-specific electrostatic interactions with actin^40^—and increases macrophage phagocytosis^41^. In addition, knockout of an LL-37 analogue in mice (*Cnlp*^*−/−*^) diminished bacterial phagocytosis^41^. We recently reported that TAGLN2 is highly expressed in macrophages upon LPS stimulation and plays an essential role in macrophage phagocytosis via controlling membrane ruffling^3^. Thus, it would be worthwhile to investigate whether LL-37 controls macrophage membrane protrusion and small filopodia formation during phagocytosis. Along this line, SipA protein, which is one of the components of bacterially encoded type III protein secretion system (TTSS)−1^42,43^, also extensively polymerizes G-actin *in vitro* and triggers large-scale membrane protrusions and ruffles at the site of *Salmonella* entry^42,44^. Although further investigations are required, these results strongly imply that proteins that drive G-actin polymerization in low-salt conditions may induce membrane ruffling as well as small filopodia formation.

EM-based structural analysis of the TAGLN2–actin complex revealed that TAGLN2 binds to actin and stabilizes actin filaments as we previously proposed^2^. This suggested that the ABPs that belong to this family may have the potential to block the binding of the Arp2/3 complex to actin if they share a binding site with the Arp2/3 complex. One example is the actin-stabilizing protein EPLIN, which binds and sequesters actin filaments and therefore prevents secondary activation of nucleation by blocking the Arp2/3 complex^25^. Although it is not clear whether EPLIN also localizes to the filopodia tips and controls their extension, further experiments will be necessary to clarify the relationship between the actin-stabilizing proteins and their ability to induce filopodia formation. However, these findings raise an important question: Why do cells require the ABPs in this criterion in addition to general Arp2/3 inhibitors? The answer probably lies in the diversity of functions performed by actin cytoskeletons, as more diverse actin structures may be required to carry out normal functions in a variety of microenvironments. In this regard, competition between Arp2/3 complex and actin side-binding proteins such as TAGLN2 and EPLIN for F-actin binding would be much more efficient for cells to generate actin filaments resistant to Arp2/3 binding in order to quickly construct actin bundles for filopodia, rather than developing another mechanism to only suppress Arp2/3 activity.

Although the Arp2/3 complex is important for filopodial initiation, it seems to be dispensable for subsequent filopodial maintenance. Therefore, this complex must be excluded from the shafts of filopodia in cells. However, the mechanism by which the Arp2/3 complex is excluded from the filopodial initiation site remains a mystery. It is also unknown how actin can be polymerized without actin branching under the condition of Arp2/3 complex activation. Formin members (or their non-mammalian homologs) are primary candidates for involvement in the elongation of filopodia^27^. This is based on the tip nucleation model, in which formins play important roles in nucleating and elongating actin filaments as well as protecting them from capping and cross-linking. However, accumulating evidence has demonstrated that filopodial actin filaments are initiated across the lamellipodium, not at a focal point as predicted by the tip nucleation model, and then gradually converge during elongation^27^. These facts support the convergent elongation model, in which the Arp2/3 complex is important for filopodial initiation and emergence from the lamellipodial dendritic network. Selective blocking of Arp2/3 complex binding to actin may be the mechanism by which the Arp2/3 complex is excluded from filopodia. In this regard, the action mechanism of TAGLN2 fits well with the convergence elongation model. Therefore, TAGLN2 may be important in microenvironments where both bundled and branched F-actin are required, as observed in T-cell spreading, neuronal growth cones, and at the leading edge of migrating epithelial cells.

In summary, a schematic model of the potential mechanism of TAGLN2 in inducing filopodial membrane protrusion is shown in Supplementary Fig. S4. In this model, TAGLN2 acts at two points. First, it can participate at the initial point of filament elongation. At this point, recruited TAGLN2 competes with the Arp2/3 complex, thereby excluding the Arp2/3 complex from the precursors of filopodia. Second, once filopodia are formed, TAGLN2 adhered to one actin filament can recruit another adjacent actin filament to elongate the shafts of filopodia, ensuring the concentration of TAGLN2 is high enough, which is necessary for coordination of actin elongation and bundling at the filopodial tips. Several factors that regulate filopodia formation have been identified *in vivo*. However, we here add new evidence that TAGLN2 belongs to the protein family that induces small filopodia formation in various cell types. This mechanism is potentially due to the biochemical characteristics of TAGLN2, which induces G-actin polymerization and competes with Arp2/3 complex for actin binding.

## Materials and Methods

### Reagents and antibodies

Rabbit polyclonal anti-TAGLN2 antibody was generated in rabbits using purified full-length TAGLN2 (AbFrontier, Seoul, Korea). The following purchased antibodies were used: goat polyclonal anti-TAGLN1 (Santa Cruz Biotechnology, Dallas, TX, USA); rabbit polyclonal anti-β-actin, HRP-conjugated anti-mouse IgG, anti-goat IgG, and anti-rabbit IgG (Cell Signaling Technology, Danvers, MA, USA); mouse monoclonal TAGLN3 and Arp3 (Abcam, Cambridge, UK); and anti-mouse CD28 (R&D Systems, Minneapolis, MN, USA). TRITC-phalloidin, poly-l-lysine (PLL), fibronectin, and phorbol 12-myristate 13-acetate (PMA) were purchased from Sigma (St. Louis, MO, USA). The mouse anti-CD3 hybridoma cell line 145–2C11 was purchased from the American Type Culture Collection (ATCC; CRL-1975, Manassas, VA, USA). The anti-human ICAM1 hybridoma R6.5 cell line was a gift from Dr. Timothy A. Springer (Harvard University, Cambridge, MA, USA). Staphylococcal enterotoxin E (SEE) and staphylococcal enterotoxin B (SEB) were obtained from Toxin Technology, Inc. (Sarasota, FL, USA). Lipofectamine 2000 transfection reagent was purchased from Life Technologies (Carlsbad, CA, USA). TAGLN2-targeting and scrambled control siRNAs were obtained as a pool of four siRNA duplexes from Dharmacon (Lafayette, CO, USA). Atto488-actin and NEM-myosin II were obtained from Hypermol (Bielefeld, Germany). Arp2/3 and VCA protein were purchased from Cytoskeleton Inc. (Denver, CO, USA).

### Cells

Jurkat T (TIB-152), A7r5 (CRL-1444), HEK293T (CRL-1573), COS-7 (CRL1651), and HeLa (CCL2) cells were maintained in RPMI-1640 or DMEM (Invitrogen, Carlsbad, CA, USA) supplemented with 10% (v/v) FBS (Invitrogen). Mouse CD3^+^ T cells were purified from the mouse spleen and lymph nodes on a T-cell enrichment column (R&D Systems). To generate mouse T cell blasts, CD3^+^ T cells were incubated in 2 µg/mL anti-CD3-coated coverslips with 2 µg/mL anti-CD28 and 10 µg/mL rIL-2 for 48 h.

## Animals

TAGLN2-knockout mice were described previously^2^. All mice were housed in specific pathogen-free conditions. All experimental methods and protocols were approved by the Institutional Animal Care and Use Committee of the School of Life Sciences, Gwangju Institute of Science and Technology and carried out in accordance with their approved guidelines (IACUC GIST-2015–04).

### cDNA constructs

The pEGFP-N1, pcDNA3.1 (CMV promoter; Clontech, Mountain View, CA, USA), and modified pHJ-1 lentiviral (CMV promoter) vectors encoding TAGLN2 were described previously^2^. TAGLN2ΔN (Δ1–24) and TAGLN2ΔCR (Δ174–199) were generated by standard PCR, whereas TAGLN2ΔH1 (Δ154–161) and TAGLN2ΔAB (Δ153–160) were generated by overlapping PCR. The 69- and 477-bp products for TAGLN2ΔH1 or 408- and 81-bp products for TAGLN2ΔAB from first-round PCR were used as overlapping templates to generate TAGLN2ΔH1 and TAGLN2ΔAB. To produce His-tagged TAGLN2 (His-TAGLN2), TAGLN2ΔN, TAGLN2ΔAB, and TAGLN2ΔCR recombinant proteins, the PCR-amplified cDNAs encoding WT or mutant TAGLN2 were ligated into the pET-28a or pGEX4T-1 vector. The Lifeact-RFP construct was a gift from Dr. Woo Keun Song (GIST).

### Cell transfection and lentiviral infection

Jurkat T-cell transient transfections were mostly performed by electroporation using the human T-cell Nucleofactor and Nucleofactor Kit V (Lonza, Basel, Switzerland). COS-7 and HeLa cell transfections were performed using Lipofectamine 2000. To establish stable cell lines, cDNAs in pHJ-1 vector were cotransfected with lentiviral packaging vectors into HEK-293T-cells. After 48 h, the supernatants were collected and spin-infected into COS-7 cells by centrifugation at 800 × g for 90 min in the presence of polybrene (8 μg/mL). For the siRNA-mediated knockdown of TAGLN2, 150 μM siRNAs were introduced into target cells and cultured for 48 h before use in experiments.

### Western blot analysis

Cells were lysed in ice-cold lysis buffer (50 mM Tris-HCl pH 7.4, 150 mM NaCl, 1% Triton X-100, 1 × complete protease/phosphatase inhibitor cocktail) for 1 h on ice. Cell lysates were centrifuged at 16,000 × g for 25 min at 4 °C, and the harvested supernatants were mixed with SDS sample buffer (100 mM Tris-HCl pH 6.8, 4% SDS, 20% glycerol, bromophenol blue) and then heated for 5 min. Proteins were separated with 10–12% SDS-PAGE gels and transferred to nitrocellulose membranes using a Trans-Blot SD Semi-Dry transfer cell (Bio-Rad, Hercules, CA, USA). Membranes were then blocked in 5% skim milk for 1 h, rinsed, and incubated with primary antibodies in TBS containing 0.1% Tween 20 (TBS-T) and 3% milk overnight. Excess primary antibody was removed by washing the membrane four times in TBS-T before incubation with peroxidase-labelled secondary antibody (0.1 μg/mL) for 1.5 h. Bands were visualised with a Western Blot Detection Kit (Intron Biotechnology, Seongnam, Korea) and exposed to X-ray film.

### Confocal and TIRF microscopy

COS-7 cells were placed on a coverglass (SPL, tissue-culture treated) and stimulated with 500 nM PMA for 15 min. HeLa cells were seeded on fibronectin (10 μg/mL)-coated coverslips for 1 h at 37 °C. For actin staining, cells were fixed with 4% PFA, permeabilized with Triton X-100 in PBS (PBS-T) for 10 min, and then incubated with TRITC-phalloidin in PBS for 30 min at room temperature. HEPES buffer was used in place of PBS for live-cell imaging. Images were obtained using a 100 × NA 1.40 oil immersion objective and an FV1000 laser scanning confocal microscope (Olympus, Tokyo, Japan). Filopodial parameters, lengths, and numbers were assessed using the software FiloQuant of ImageJ (National Institutes of Health, Bethesda, MD, USA). To monitor actin polymerization in real time, Atto488-actin subunits were centrifuged at 200,000 × g for 20 min, and the supernatants were used in experiments. Ca-actin was converted to Mg-actin for each experiment by adding 10 × Mg exchange buffer (10 mM EDTA and 1 mM MgCl_2_) at 1:10 and incubating on ice for 2 min. NEM-myosin II was diluted to 0.2 μM in high-salt Tris-buffered saline (HS-TBS, 50 mM Tris-HCl pH 7.6 and 600 mM NaCl) and loaded onto a coverglass. After 2 min, the coverglass was washed with 1% (w/v) bovine serum albumin (BSA) in low-salt Tris-buffered saline (LS-TBS, 50 mM Tris-HCl pH 7.6 and 50 mM NaCl). Mg-ATP-actin (2×) was mixed 1:1 with 2 × F-buffer (100 mM KCl, 2 mM MgCl_2_, 2 mM EGTA, 20 mM imidazole pH 7.0, 100 mM DTT, 0.4 mM ATP, 30 mM glucose, 1% methylcellulose, 40 μg/mL catalase, and 200 μg/mL glucose oxidase), and 10 μL was immediately loaded onto the coverglass, which was placed on the microscope. For polymerization in G-buffer, KCl, MgCl_2_, and EGTA were excluded from the F-buffer. In all polymerization analyses, 0.2 μM Atto488-actin and 0.4 μM TAGLN2 or TAGLN2 mutant proteins were premixed with Mg-actin for 3 min. VCA (50 nM) and Arp2/3 (2.5 nM) were used for inhibitory assays of actin nucleation. Images were recorded every 10 s for a total 1,200 s. To quantify the length of fluorescent actin filaments, individual filaments (n = 200) in acquired images were measured by careful manual tracing using the Olympus-Fluoview program. To visualize the actin growth from the F-T/actin seed, Mg^2+^-exchanged Ca^2+^-Atto594 actin (0.2 μM) was mixed with TAGLN2 protein (0.4 μM) for 3 min in G-buffer condition, diluted 1/10 in G-buffer, and loaded onto an NEM-coated coverglass. After 5 min, the coverglass was washed with 1% BSA in LS-TBS. The Mg^2+^-exchanged Ca^2+^-Atto488 actin (0.2 μM) mixture in F-buffer was then loaded onto the coverglass.

To analyse TAGLN2 localization at the immunological synapse, Jurkat T cells expressing GFP or TG2 (TAGLN2)_GFP were mixed with SEE-loaded Raji B cells stained with Cy3-conjugated ICAM-1, pre-incubated for 20 min, and placed on PLL-coated coverslips. Images were acquired with the FV1000 laser scanning confocal microscope. Mouse CD3^+^ T cells from WT and *TAGLN2*^*−/−*^ mice were incubated with anti-CD3 (10 μg/mL)/CD28 (2 μg/mL) for 10 min. Actin staining was performed using the method described in the previous section. Images were obtained using structured illumination microscopy (SIM).

### Purification of recombinant proteins

Recombinant protein expression in *Escherichia coli* BL21 (DE3) cells was performed following the protocol used for transformation of recombinant plasmids. The expression of recombinant proteins was induced by the addition of IPTG (0.3 mM) to culture media for 4 h at 37 °C. Harvested cells were resuspended in PBS, sonicated, and centrifuged. After centrifugation, recombinant proteins in the supernatant were purified by affinity chromatography on a His-selected Nickel Affinity Gel (Sigma) equilibrated with 10 volumes of buffer (50 mM sodium phosphate pH 8.0 and 0.3 M NaCl). Equilibrated gels were incubated with the protein supernatant and then washed with 5 volumes of wash buffer (50 mM sodium phosphate pH 8.0, 0.3 M NaCl, and 10 mM imidazole). The bound protein was eluted with increasing concentrations of imidazole up to 250 mM, the collected fractions were dialysed to remove imidazole, and the purified proteins were assessed by SDS-PAGE and Coomassie blue staining. In some experiments, thrombin was incubated with His_TG2 on the column overnight at 4 °C.

### Actin polymerization assays

Actin polymerization assays were performed using the Actin Polymerization Biochem Kit (Cytoskeleton). Briefly, pyrene-labelled G-actin was thawed at 4 °C, centrifuged at 100,000 × g for 30 min at 4 °C to remove residual filamentous actin, and then mixed in G-buffer (0.2 mM CaCl_2_, 0.2 mM ATP, and 5 mM Tris-HCl pH 7.5) to produce an actin stock. To induce polymerization in G-buffer, 4 μM actin (50% actin monomers) was incubated with increasing concentrations of His-TAGLN2 (His-TG2; 2, 4, 8, 16, and 32 μM) in G-buffer. To polymerize in F-buffer, 2 μM actin (50% actin monomers), bovine brain Arp2/3 complex (20 nM), recombinant human GST-tagged WASp VCA domain (200 nM), and increasing concentrations of His-TG2 (0, 2, 4, 8, 16, and 32 μM) were incubated in F-buffer (50 mM KCl, 2 mM MgCl_2_, 0.2 mM ATP, and 5 mM Tris-HCl pH 7.5) unless otherwise indicated. The plate was read with a Victor X3 (Perkin Elmer, Wellesley, MA, USA) with excitation and emission wavelengths of 355 nm and 405 nm, respectively. Actin polymerization was measured as arbitrary fluorescence intensity (in arbitrary units [a.u.]) over time (s). For halftime (t_1/2_) calculations, G-actin polymerization assays with increasing concentrations of TAGLN1, 2, or 3 were performed, and the time was calculated at half-maximal pyrene intensities.

### Actin depolymerization assay

For monitoring actin filament disassembly, pyrene G-actin (20 μM) was polymerized in APB for 1 h in the presence or absence of various concentrations of TAGLN2 or phalloidin (20 μM, Cytoskeleton). Reactants were then diluted to 4 μM with G-actin buffer to induce spontaneous depolymerization. In the case of cofilin-mediated depolymerization, cofilin (1 μM, Cytoskeleton) in G-buffer was added to F-actin reactants during the dilution step. Decreasing pyrene fluorescence was measured as in the actin polymerization assay. Normalization for fluorescence intensity was performed by dividing the fluorescence value of reactants by the fluorescence value of the reactant in the presence of phalloidin at each time point. Changes in fluorescence intensity (ΔF) during depolymerization were calculated by subtracting the fluorescence measured at the last time point from the fluorescence measured at the initial time point. Changes of each sample (ΔF of TAGLN2 or TAGLN2 + cofilin) were subsequently divided by ΔF of cofilin.

### *In vitro* actin co-sedimentation assays

Actin co-sedimentation assays were performed as described previously^45^. Muscle actin derived from rabbit smooth muscle was obtained from Cytoskeleton. ATP-actin was used at a final concentration of 4 μM. ATP-actin was mixed in G-buffer (0.2 mM CaCl_2_, 0.2 mM ATP, and 5 mM Tris-HCl pH 7.5) to produce an actin stock. ATP actin was polymerized in G-buffer with His-TG2 or TG2ΔAB or in F-buffer at room temperature for 30 min. Actin filaments with bound proteins were pelleted by centrifugation at 200,000 × g for 1 h (for the F-actin binding assay) at room temperature. Equal amounts of pellet (p) and supernatant (s) fractions were resolved by SDS-PAGE, and the proteins were visualised by Coomassie blue staining. The relative percentages of actin in supernatant and pellet fractions were quantified by densitometry using ImageJ. For Arp2/3 binding competition assay, actin (2 μM), Arp2/3 complexes (20 nM), and VCA domain of WASp (200 nM) were incubated with increasing concentrations of TAGLN2 or TG2ΔAB (0, 2, 4, 8 μM) for 30 min. Actin filaments with bound proteins were pelleted by centrifugation at 200,000 × g for 1 h at room temperature. Equal amounts of pellet (p) and supernatant (s) fractions were resolved using SDS-PAGE, followed by western blotting with anti-Arp3 antibody.

### Quantification of binding data

Quantitation of actin-binding affinity was performed as previously described^45^. Briefly, the intensity ratio of recombinant protein to G-actin in each pellet was converted to a molar ratio (moles of His_TG2/moles of actin or BSA) using standard curves run on each gel that contained known amounts of His_TG2 (1–15 µM) and actin (4 µM) in mol/mol ratios. Co-sedimentation binding data were plotted as a function of the free TAGLN2 concentration added and fitted according to the equation: [C_bound_]/[Actin] = B_max_[C_free_]/*K*_d + _[C_free_], where [C_bound_] and [C_free_] are the bound and free concentrations of TAGLN2, [Actin] is the total actin concentration, B_max_ is the maximal molar binding ratio, and *K*_d_ is the dissociation constant (μM). Data was fit using the Microsoft Excel Solver package by varying the values of B_max_ and *K*_d_ and minimizing the sum of squares between the actual and predicted binding ratios.

### G-actin binding assay

G-actin binding assay was performed using the Actin-Toolkit G-Actin Binding Kit (Hypermol). Briefly, the actin beads were reconstituted in G-buffer (0.2 mM CaCl_2_, 0.2 mM ATP, and 5 mM Tris-HCl pH 7.5) and incubated with His_TG2 (1–10 μg) for 60 min at room temperature. Gelsolin (2.5 μg) and actin (2.5 μg) were used as a positive and negative control, respectively. Actin beads were washed three times with G-buffer. Proteins bound to actin beads were released by boiling, subjected to SDS-PAGE, and visualized by Coomassie blue staining.

### Electron microscopy and 3D reconstruction

For scanning EM, cells were fixed with 2.5% glutaraldehyde solution for 2 h, rinsed with PBS for 5 min, and fixed in OsO_4_ for 2 h. Samples were then dehydrated through incubation with a graded ethanol series over 30 min and dried in a critical point dryer. Samples were prepared by sputter coating with 1–2 nm gold-palladium and analysed using FE-SEM (HITACHI, Tokyo, Japan). The number of finger-like structures was quantified by counting in a 10 × 10-μm area. Finger-like structures were defined as filaments protruding >1.5 μm from the main shaft. For transmission EM, 2 μM F-actin and 4 μM TAGLN2 were mixed together in buffer. Samples were incubated at room temperature for 30 min. Aliquots (7 μL) were then applied to EM grids coated with thin carbon supported by a holey carbon film and negatively stained with 1% uranyl acetate^46^. Dried grids were observed with a Tecnai 12 electron microscope (FEI, Hillsboro, OR, USA) at 120 kV under low-dose conditions. Images of filaments were acquired by a 4 K × 4 K CCD camera (Eagle, FEI). Long, relatively straight filaments were unbent using ImageJ and converted to SPIDER format (EM2EM; Image Science and Imperial College, London, UK). Selected filaments were cut into segments in SPIDER^47^, and iterative helical real space reconstruction (IHRSR)^46^ was carried out using SPIDER. An F-actin model was used as an initial reference for the first round of IHRSR. UCSF Chimera^48^ was used for visualization, analysis, and atomic fitting of 3D volumes. Samples for platinum replica EM were processed essentially as described previously^49^.

### Statistics

Mean values were calculated from at least three independent experiments using unpaired Student’s *t*-tests, and one-way analysis of variance (ANOVA) tests were used to identify significant differences between groups (*p* < 0.05).

## Electronic supplementary material

Supplementary video S1

Supplementary video S2

Supplementary video S3

Supplementary video S4

Supplementary video S5

Supplementary video S6

Supplementary information
