# Supplementary material for: TAGLN2 polymerizes G-actin in a low ionic state but blocks Arp2/3-nucleated actin branching in physiological conditions
Source: Sci Rep. 2018 Apr 3;8:5503. doi: 10.1038/s41598-018-23816-2 (PMC5883021; doi:10.1038/s41598-018-23816-2)
Supplement: Supplementary file 7 — Supplementary information [file 41598_2018_23816_MOESM7_ESM.pdf]

Supplementary Materials for

TAGLN2 polymerizes G-actin in a low ionic state but blocks Arp2/3-nucleated actin branching in physiological conditions

Hye-Ran Kim[1,2], Min-Sung Kwon[1,3], Sangmin Lee[4], YeVin Mun[1,2], Kyung-Sik Lee[1,2], Chang-Hyun Kim[1,2], Bo-Ra Na[1,2], Bit Na Rae Kim[1], Indre Piragyte[1,2], Hyun-Su Lee[1,2], Youngsoo Jun[1], Mi Sun Jin[1], Young-Min Hyun[5], Hyun Suk Jung[4], Ji Young Mun[6,*] & Chang-Duk Jun[1,2,*]

This PDF file includes:

Figs. S1 to S9

Figure legends for Figs. S1 to S4

Captions for Videos 1 to 6

Figure S1. 6×His tag does not affect the actin polymerization activity of TAGLN2.

(a) Purification of TAGLN2 by treatment with thrombin. C, crude fraction; E1, eluted fraction with imidazole; E2, fraction of thrombin-incubated with His-TAGLN2 at 4°C overnight; His-TAGLN2 (25 kDa) and TAGLN2 (22 kDa). (b) Time-based fluorometric analysis of pyrene-labelled actin polymerization. Comparison of His-TAGLN2 with TAGLN2 in low-salt G-buffer by increasing concentrations (T/A = TG2/actin).

Figure S2. Sequence alignment and secondary structure of TAGLN1, 2, and 3.

Schematic diagram represents structure-based sequence alignment of TAGLN family members. Blue and red boxes indicate residues showing positive and negative charges, respectively. Potential phosphorylation sites by *in silico* prediction are marked with an asterisk.

Figure S3. Structure comparison of human TAGLN2 and *Salmonella* SipA

(a) Full-length human TAGLN2 and *Salmonella* SipA are represented schematically. (b) The CH domain (PDB ID: 1WYM) of human TAGLN2 is superimposed with the N-terminal (PDB ID: 2FM9) and (c) C-terminal actin binding (PDB ID: 1Q5Z) domains of *Salmonella* SipA. The structures were downloaded from the protein data bank.

Figure S4. Schematic model of the roles of TAGLN2 in filopodium formation.

(1) At the leading edges of lamellipodia, where the branched actin network is formed by Arp2/3 complex-mediated nucleation, recruited TAGLN2 competes with the Arp2/3 complex to bind to actin filaments, thereby excluding Arp2/3 complexes from the precursors of microvilli. (2) Once microvilli are initiated, actin filaments with TAGLN2 can be cross-

linked with adjacent filaments, presumably in some cases by other bundling proteins, to create a new microvillus shaft.

Supplemental Movies

Movie S1. Spontaneous polymerization of non-muscle actin filament (0.2 μM, Atto488-labelled) on myosin II-coated coverslips in low-salt buffer (1.46 MB, avi).

Movie S2. Spontaneous polymerization of non-muscle actin filament (0.2 μM, Atto488-labelled) on myosin II-coated coverslips in the presence of TAGLN2 (0.4 μM) in low-salt buffer. G-actin and TAGLN2 were pre-incubated together before the addition of G-buffer (1.85 MB, avi).

Movie S3. Spontaneous polymerization of non-muscle actin filament (0.2 μM, Atto488-labelled) on myosin II-coated coverslips in high-salt buffer (1.86 MB, avi).

Movie S4. Spontaneous polymerization of non-muscle actin filament (0.2 μM, Atto488-labelled) on myosin II-coated coverslips in the presence of TAGLN2 (0.4 μM) in high-salt buffer. G-actin and TAGLN2 were pre-incubated together before the addition of F-buffer (1.86 MB, avi).

Movie S5. Actin nucleation of non-muscle actin filament (0.2 μM, Atto488-labelled) in the presence of 2.5 nM Arp2/3 and 50 nM VCA on myosin II-coated coverslips in high-salt buffer (1.73 MB, avi).

Movie S6. Inhibition of actin nucleation of non-muscle actin filament (0.2 μM, Atto488-labelled) by TAGLN2 in the presence of 2.5 nM Arp2/3 and 50 nM VCA on myosin II-coated coverslips in high-salt buffer (1.70 MB, avi).

Figure S1

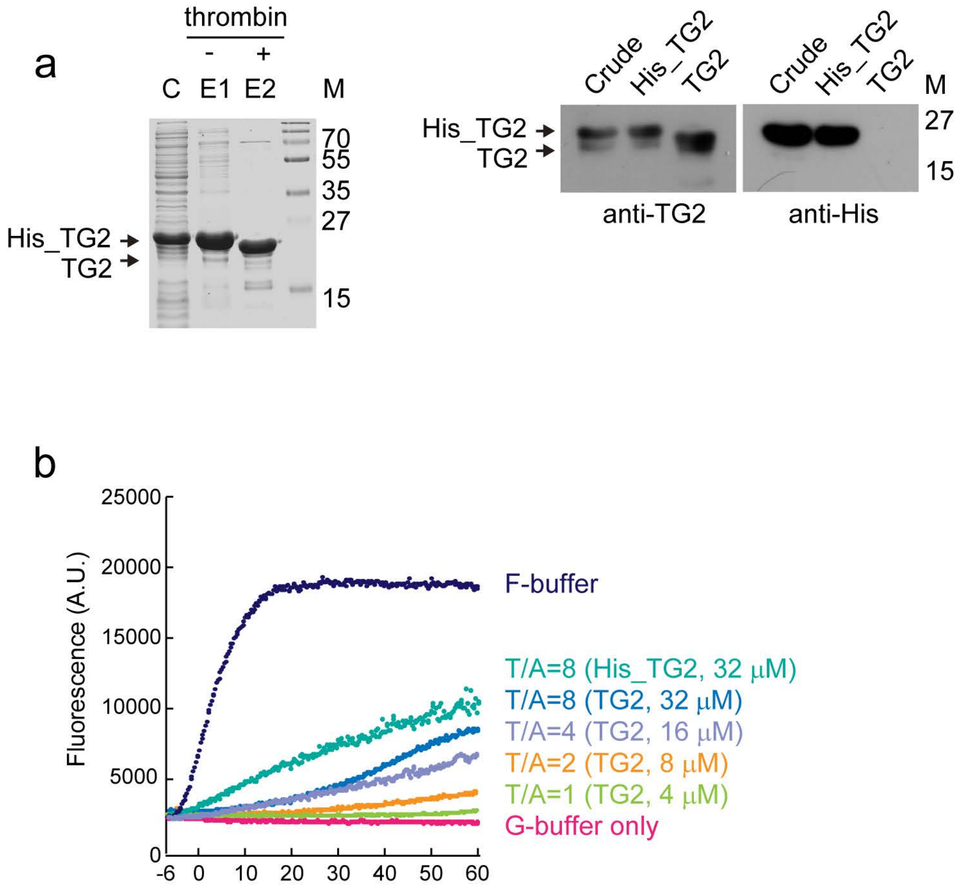
a
thrombin
-
+
C
E1
E2
M
70
55
35
27
15
His_TG2
TG2
Crude
His_TG2
TG2
Crude
His_TG2
TG2
M
27
15
His_TG2
TG2
anti-TG2
anti-His
b
Fluorescence (A.U.)
25000
20000
15000
10000
5000
0
-6
0
10
20
30
40
50
60
F-buffer
T/A=8 (His_TG2, 32 μM)
T/A=8 (TG2, 32 μM)
T/A=4 (TG2, 16 μM)
T/A=2 (TG2, 8 μM)
T/A=1 (TG2, 4 μM)
G-buffer only

Figure S2

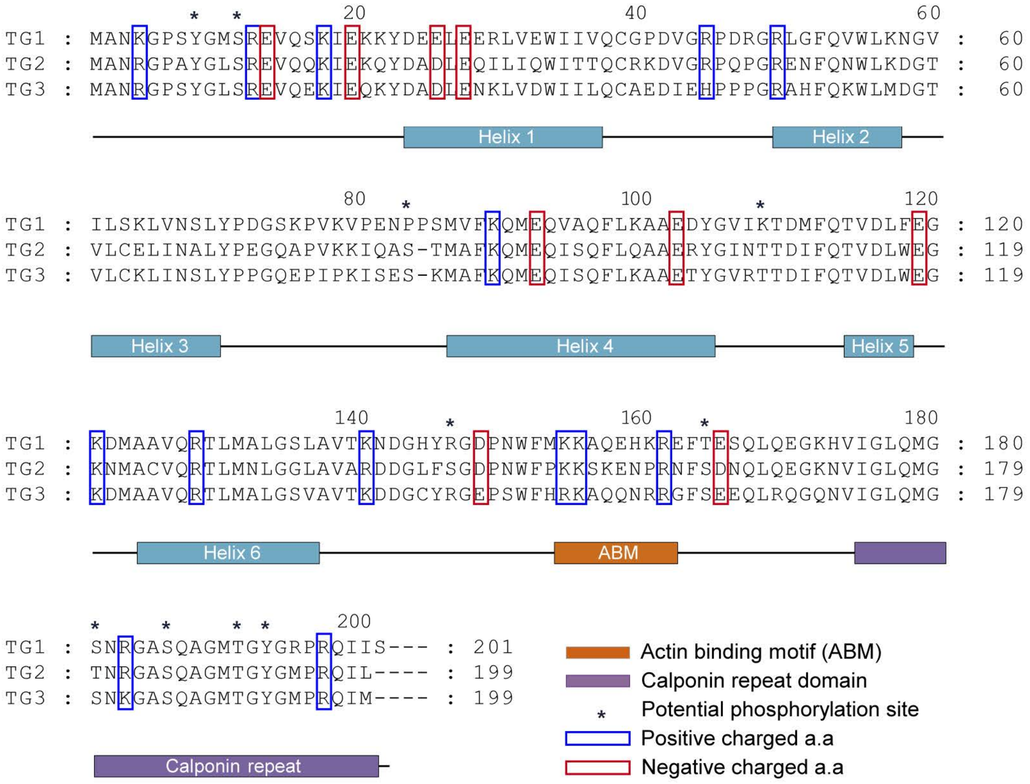
20 40 60
TG1 : MANKGPSYGMSREVQSKIEKKYDEEIEERLVEWIIVQCGPDVGRPDRGRLGFQVWLKNGV : 60
TG2 : MANRGPAYGLSREVQQKIEKQYDADLEQILIQWITTQCRKDVGRPQPGRENFQNWLKDGT : 60
TG3 : MANRGPSYGLSREVQEKIEQKYDADLENKLVDWIILQCAEDIEHPPPGRAHFQKWLMDGT : 60
Helix 1
Helix 2
80 100 120
TG1 : ILSKLVNSLYPDGSKPVKVPENPPSMVFKQMEQVAQFLKAAEDYGVIKTDMFQTVDLFEG : 120
TG2 : VLCELINALYPEGQAPVKKIQAS-TMAFKQMEQISQFLQAAERYGINTTDIFQTVDLWEG : 119
TG3 : VLCKLINSLYPPGQEPIPKISES-KMAFKQMEQISQFLKAAETYGVRTTDIFQTVDLWEG : 119
Helix 3
Helix 4
Helix 5
140 160 180
TG1 : KDMAAVQRTLMALGSLAVTKNDGHYRGDPNWFMKKAQEHKREFTESQLQEGKHVIGLQMG : 180
TG2 : KNMACVQRTLMNLGGLAVARDDGLFSGDPNWFPKKSKENPRNFSDNQLQEGKNVIGLQMG : 179
TG3 : KDMAAVQRTLMALGSVAVTKDDGCYRGEPSWFHRKAQQNRRGFSEEQLRQGQNVIGLQMG : 179
Helix 6
ABM
200
TG1 : SNRGASQAGMTGYGRPRQIIS--- : 201
TG2 : TNRGASQAGMTGYGMPRQIL---- : 199
TG3 : SNKGASQAGMTGYGMPRQIM---- : 199
Calponin repeat
Actin binding motif (ABM)
Calponin repeat domain
Potential phosphorylation site
Positive charged a.a
Negative charged a.a

Figure S3

a

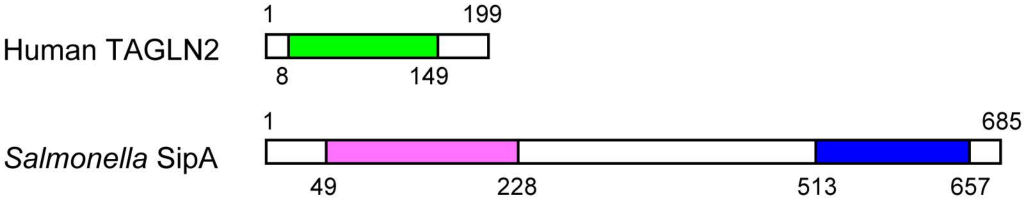

b

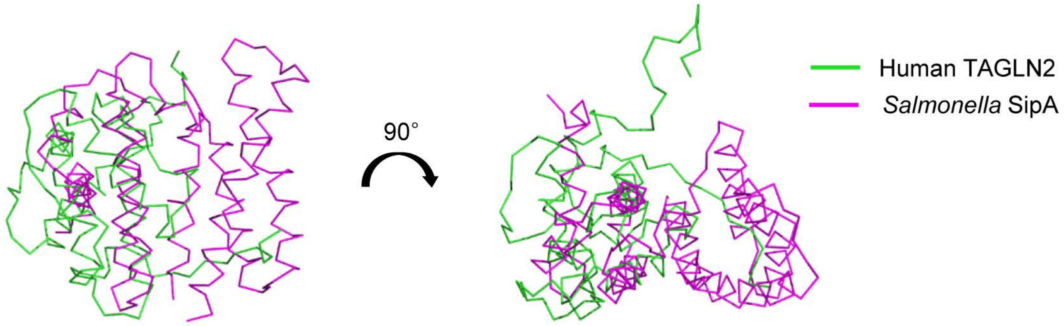


```
Human    TAGLN2      8 -----------------------------------------------YGLSREVQQKIEK 20
Salmonella SipA     49 PQLEDFPALIKQASLDALFKCGKDAEALKEVFTNSNNVAGKKAIMEFAGLFRSALNATSD 108
                                                                       ** *.. :  ..

Human TAGLN2        21 QYDADLEQILIQWITTQCRKDVGRPQPGRENFQNWLKDGTVLCELINAL--------YPE 72
Salmonella SipA    109 S--PEAKTLLMKVGAEYTAQIIKDGLKEKSAFGPWLPETKKAEAKLENLEKQLLDIIKNN 166
                       .   : : :*::  :    : :      :. *  ** : .     :: *          :

Human TAGLN2        73 GQAPVKKIQASTMAFKQMEQISQFLQAAERYGINTTDIFQTVDLWEGKNMACVQRTLMNL 132
Salmonella SipA    167 TGGELSKLSTNLV----MQEVMPYI-----------------ASCIEHNFGCTLDPLTRS 205
                         . :.*:.:. :    *:::  ::                      :*:.*.   * .

Human TAGLN2       133 --------------GGLAVARDDGLFSGDPN 149
Salmonella SipA    206 NLTHLVDKAAAKAVEALDMCHQK-------- 228
                                      .* :.::.
```

c

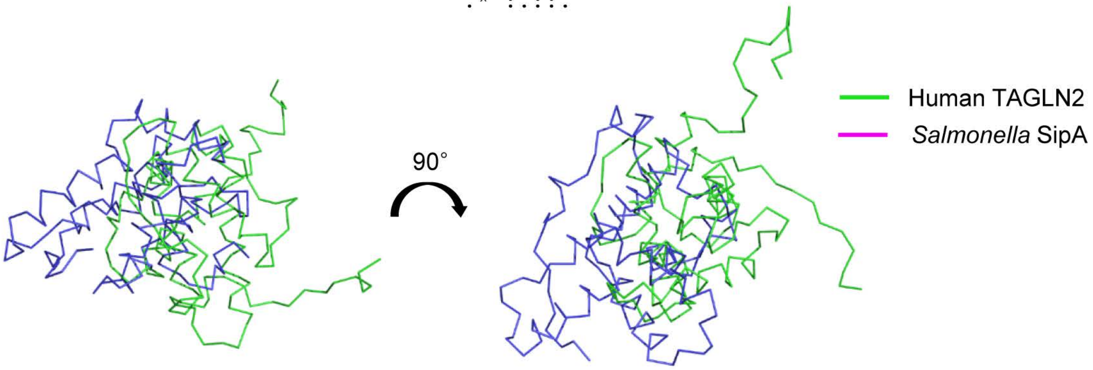


```
Human TAGLN2         8 ---YGLSR----------EVQQKIEKQYDADLEQILIQWITTQCRKDVGRPQPGRENFQN 54
Salmonella SipA    513 GPFSGLKFKQNSFLSTVPSVTNMHSMHF--DARETFLGVIRKALEPDTSTPFPVRRAFDG 570
                           **.           .* :  . ::  * .: ::  * .  . *.. * * *. *:

Human TAGLN2        55 WLKD----GTV-------LCELINALYPEGQAPVKKIQASTMAFKQMEQISQ-FLQAAER 102
Salmonella SipA    571 LRAEILPNDTIKSAALKAQCSDIDK-HPELKAKMETLKEVITHHPQKEKLAEIALQFARE 629
                          :     *:        *. *:  :** :* ::.::     . * *::::  ** *..

Human   TAGLN2     103 YGI------------NTTDIFQTVDLWEGKNMACVQRTLMNLGGLAVARDDGLFSGDPN  149
Salmonella SipA    630 AGLTRLKGETDYVLSNVLDGLIGDGSWR-------------------------------  657
                        *:            *. * :     *.
```

Figure S4

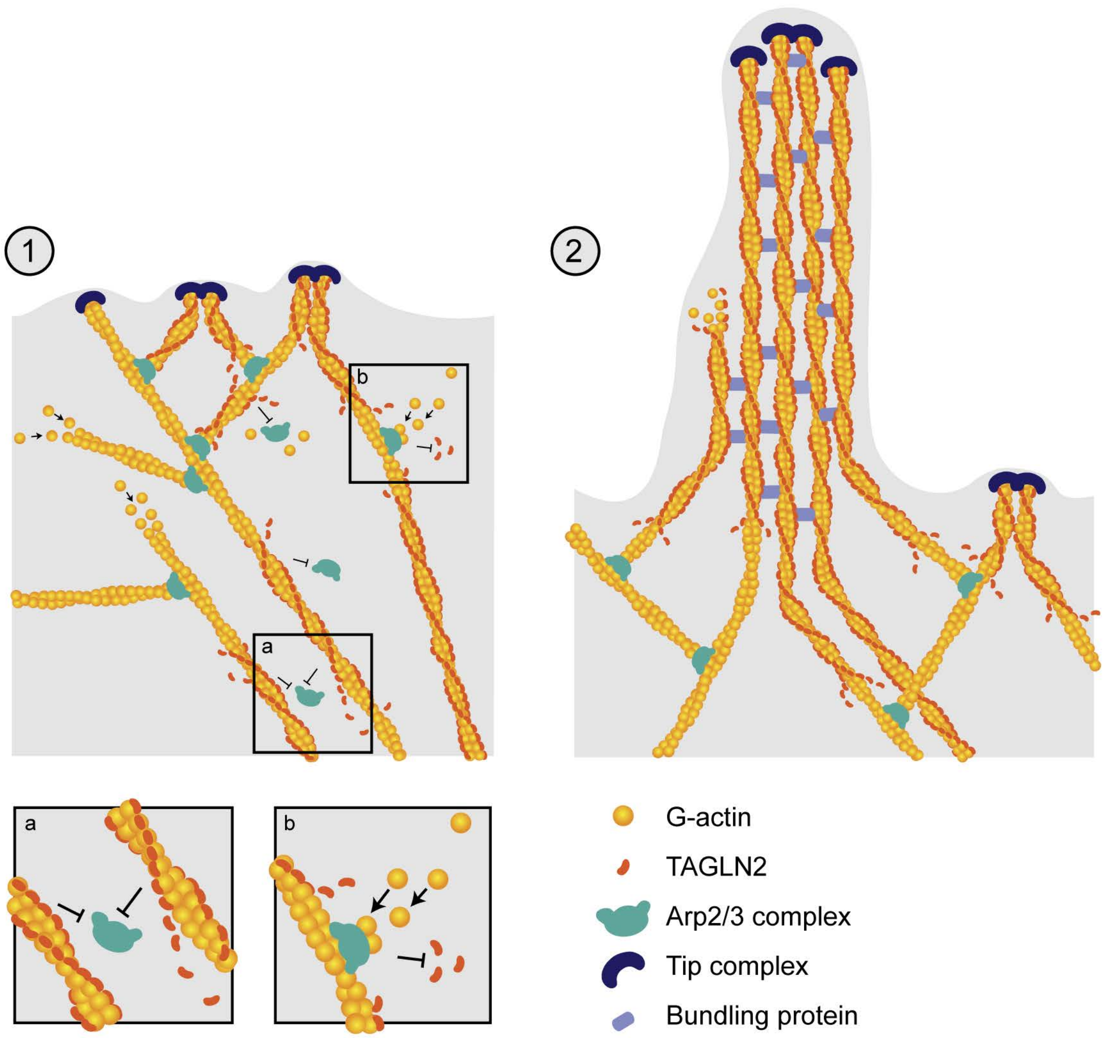

Competitive inhibition of actin-binding between TAGLN2 and Arp2/3 complex

Figure S5

Figure 1a

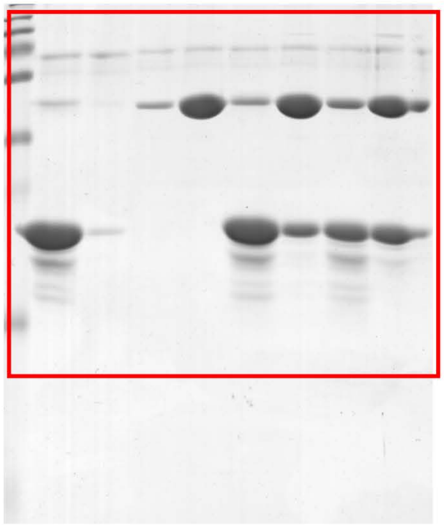

Figure 1b

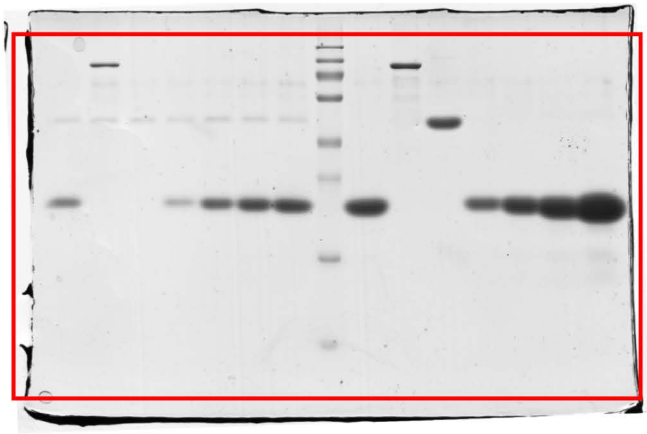

Figure 1f

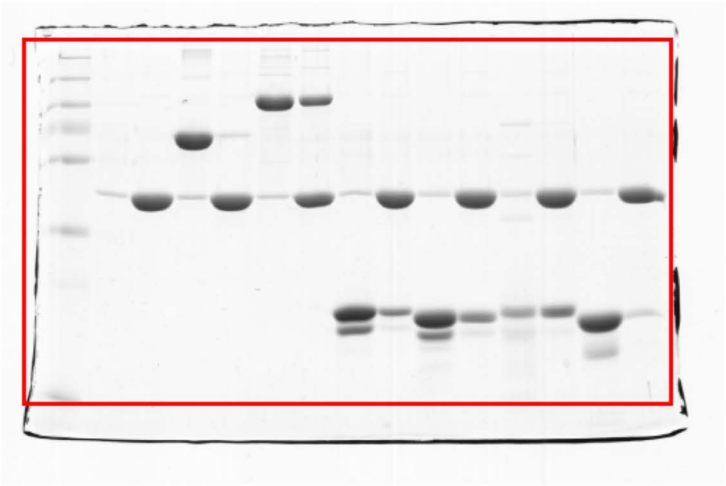

Figure S6

Figure 2b

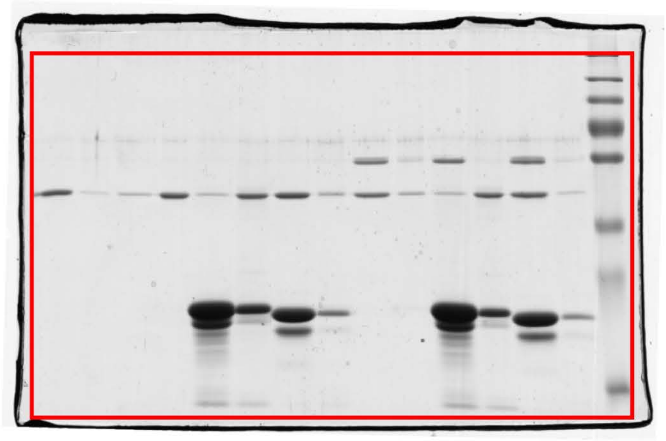

Figure 4a

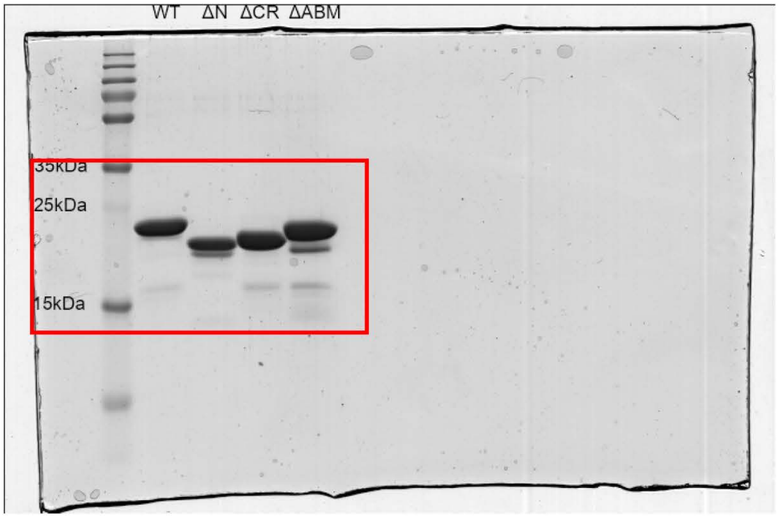

Figure S7

Figure 5d

Long exposure

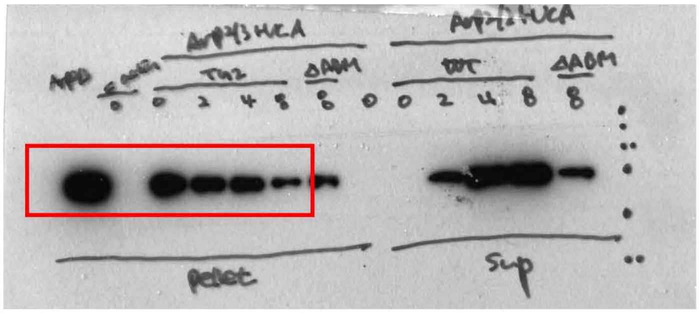

short exposure

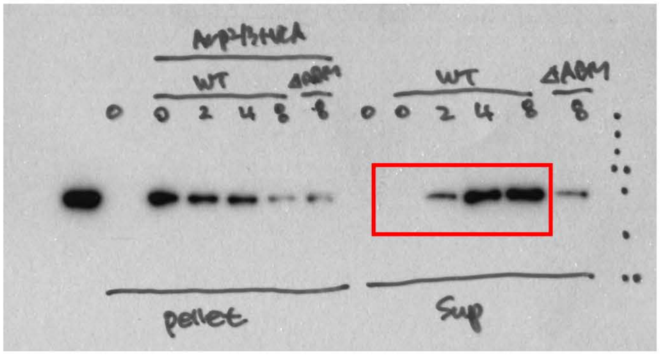

IB : anti-Arp3

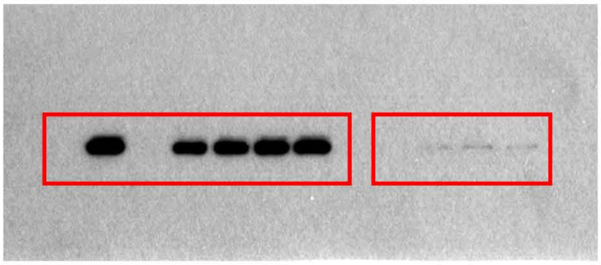

IB : anti-Arp3

Figure 6b

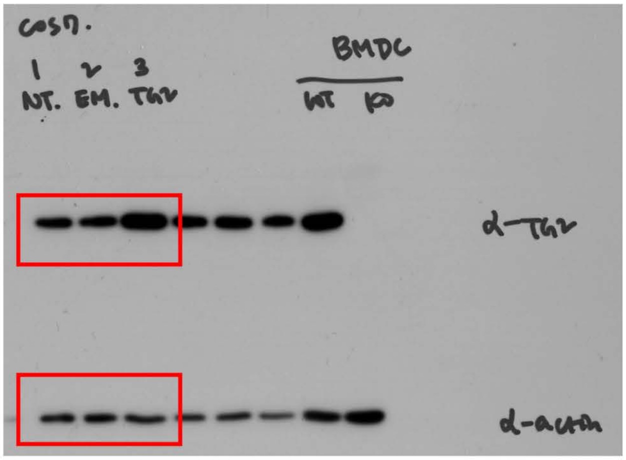

Figure S8

Figure 7a

Long exposure

short exposure

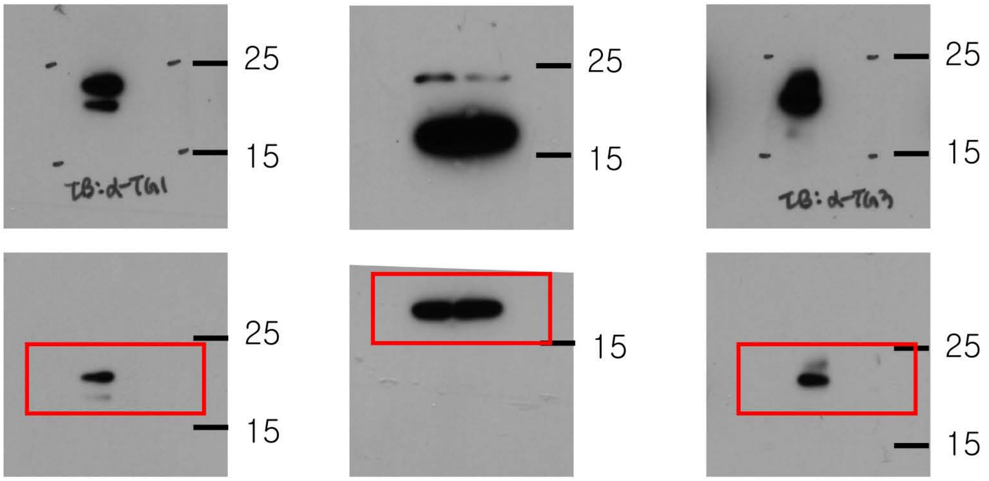

IB : anti-TG1 IB : anti-TG2 IB : anti-TG3

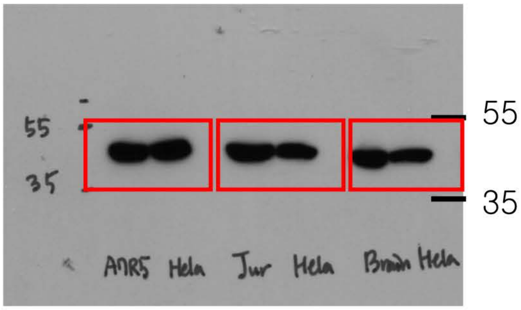

IB : anti-actin

Figure 7b

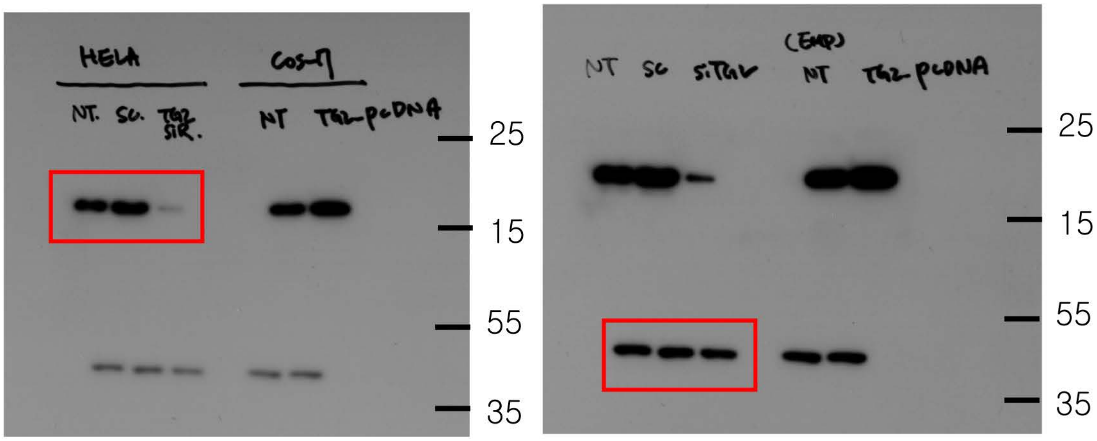

IB : anti-TG2 IB : anti-actin

Figure S9

Figure 7c

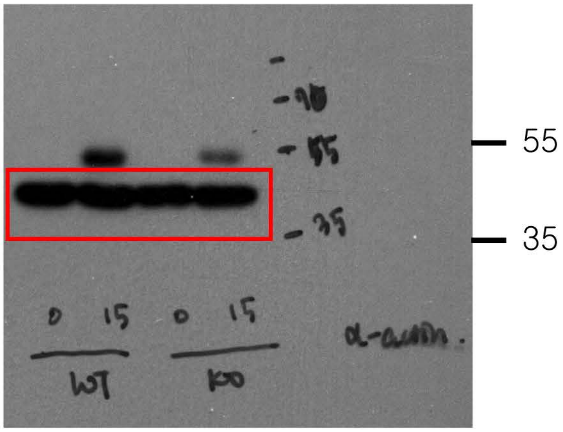

IB : anti-actin

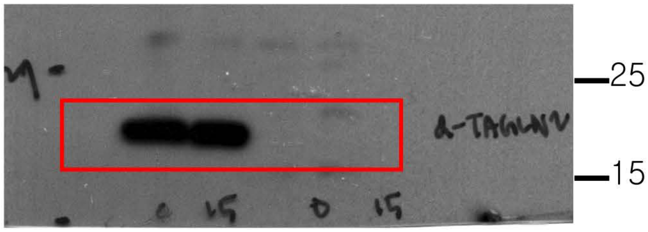

IB : anti-TAGLN2

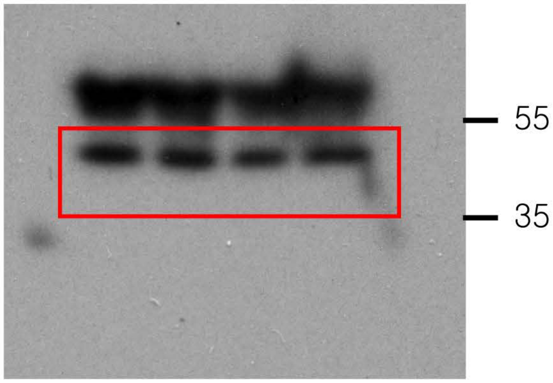

IB : anti-Arp3

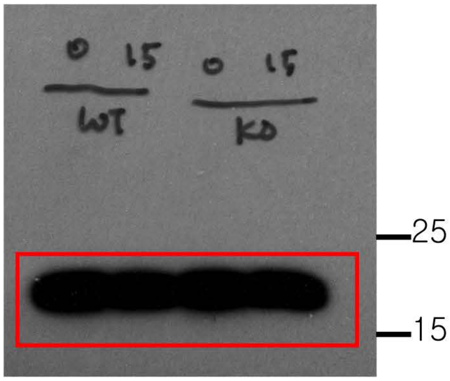

IB : anti-cofilin
